# Host and pathogen autophagy are central to the inducible local defences and systemic response of the giant kelp *Macrocystis pyrifera* against the oomycete pathogen *Anisolpidium ectocarpii*

**DOI:** 10.1111/nph.16438

**Published:** 2020-02-29

**Authors:** Pedro Murúa, Dieter G. Müller, Mohammad Etemadi, Pieter van West, Claire M. M. Gachon

**Affiliations:** ^1^ Aberdeen Oomycete Laboratory International Centre for Aquaculture Research and Development University of Aberdeen Foresterhill Aberdeen AB25 2ZD UK; ^2^ The Scottish Association for Marine Science Scottish Marine Institute Oban PA37 1QA UK; ^3^ Fachbereich Biologie der Universität Konstanz D‐78457 Konstanz Germany; ^4^ Institute of Microbiology University of Innsbruck A‐6020 Innsbruck Tyrol Austria; ^5^ UMR 7245 - Molécules de Communication et Adaptation des Micro-organismes Muséum National d'Histoire Naturelle CP 54, 57 rue Cuvier 75005 Paris France

**Keywords:** chlorophagy, lipophagy, nucleophagy, peronosporomycete, Phaeophyceae, systemic response

## Abstract

- Kelps are key primary producers of cold and temperate marine coastal ecosystems and exhibit systemic defences against pathogens. Yet, the cellular mechanisms underpinning their immunity remain to be elucidated.
- We investigated the time course of infection of the kelp *Macrocystis pyrifera* by the oomycete *Anisolpidium ectocarpii* using TEM, *in vivo* autophagy markers and autophagy inhibitors.
- Over several infection cycles, *A. ectocarpii* undergoes sequential physiological shifts sensitive to autophagy inhibitors. Initially lipid‐rich, pathogen thalli become increasingly lipid‐depleted; they subsequently tend to become entirely abortive, irrespective of their lipid content. Moreover, infected algal cells mount local defences and can directly eliminate the pathogen by xenophagy. Finally, autophagy‐dependent plastid recycling is induced in uninfected host cells.
- We demonstrate the existence of local, inducible autophagic processes both in the pathogen and infected host cells, which result in the restriction of pathogen propagation. We also show the existence of a systemic algal response mediated by autophagy. We propose a working model accounting for all our observations, whereby the outcome of the algal–pathogen interaction (i.e. completion or not of the pathogen life cycle) is dictated by the induction, and possibly the mutual hijacking, of the host and pathogen autophagy machineries.

## Introduction

Brown algae (Phaeophyceae), particularly kelps, are key primary producers and ecosystem engineers in cold and temperate coastal environments (Krumhansl *et al.*, 2016), where they impact upon atmospheric chemistry (Küpper *et al.*, 2008). Intertidal and subtidal habitats, where most brown algae live, are shaped by fluctuating environmental factors such as salinity‐, temperature‐ and light‐related stresses (reviewed by Hurd *et al.*, 2014); therefore, the responses of brown algae to abiotic stress have been comparatively well‐studied. Their importance as primary producers also has generated substantial interest in their response to grazers (Pavia *et al.*, 2012). However, the responses of brown algae to pathogens are much less well‐known, due mostly to the paucity of pathosystems amenable to physiological and genomic investigation. The available knowledge on algal responses to pathogens is therefore disproportionately concentrated on plants and other Archaeplastida (Bouarab *et al.*, 1999; Segovia, 2003; Monier *et al.*, 2017).

Pathogenic oomycetes infect a remarkable diversity of hosts ranging from brown and red algae, diatoms, molluscs, crustaceans, plants, nematodes, fungi, insects, fishes and mammals. Among them, *Anisolpidium* is a genus of obligate biotrophic pathogens that infects marine brown algae. *Anisolpidium ectocarpii* has a broad host spectrum, spanning ≥ 20 species across nine orders (Gachon *et al.*, 2017); this includes the genome model *Ectocarpus siliculosus* and the gametophytic life stages of several kelps, including of the giant Pacific kelp *Macrocystis pyrifera*. Phylogenetically, *Anisolpidium* is closely related to marine species of *Olpidiopsis*, and recently described clades of diatom pathogens named OOM_1 and OOM_2 (Garvetto *et al.*, 2018). *Olpidiopsis* is a cosmopolitan genus that encompasses devastating pathogens of red algae, including laver, the most valuable seaweed crop in the world (Klochkova *et al.*, 2016; Badis *et al.*, 2019); the OOM_1 and OOM_2 clades contain pathogens potentially relevant to the control of phytoplankton dynamics and consequently, biogeochemical cycling. Finally, the early‐diverging position of *Anisolpidium* among oomycetes (Gachon *et al.*, 2017) makes it an ideal model to address the physiology and evolutionary origin of pathogenicity and biotrophy in oomycetes. Conversely, the capacity of *A. ectocarpii* to infect a huge diversity of hosts suggests that its virulence targets are shared by many brown algae; therefore, *A. ectocarpii* also is a good model to investigate the core set of defence responses conserved across brown algae (Murúa, 2018).

In brown algae, immunological responses against pathogens are not well‐described. Elicitor recognition (by pathogen‐ or damage‐associated molecular pattern, PAMP or DAMP) and the subsequent response to pathogens, mediated for example by lipid signalling and oxidative burst, are to some extent conserved with plants and animals (Küpper *et al.*, 2001, 2009). However, genome sequencing of the model brown alga *E. siliculosus* revealed that it lacks canonical (NB‐LRR) resistance genes, and it has been suggested that its capacity to evolve new pathogen recognition specificities might stem from targeted exon shuffling within LRR‐containing GTPases and NB‐ARC‐TPR proteins (Zambounis *et al.*, 2012). How these recognition and early signalling events actually lead to defence or resistance against pathogens is virtually unknown. Additionally, brown algae are one of very few chromistan lineages that have evolved complex multicellularity, independently from plant and animals (Cock *et al.*, 2010). The existence of waterborne signalling and systemic defences has recently been demonstrated in sporophytes of the kelp *Laminaria digitata* (Thomas *et al.*, 2011, 2014), but the molecular mechanisms underpinning this inter‐ and intra‐individual communication are unknown.

Autophagy is a fundamental process in eukaryotic cells, involved in cell differentiation, homeostasis, senescence and stress responses such as nutrient deprivation. Autophagy also is key in immunity, contributing to resistance against intracellular pathogens, or conversely hijacked by pathogens, leading to the development of disease. In the haptophyte *Emiliania huxleyi*, autophagy is hijacked during infection by a virus, and it is critical to the completion of lysogeny (Schatz *et al.*, 2014). In plants and animals, autophagy can equally induce, promote, execute or inhibit cell death (Minina *et al.*, 2014); in plants, it controls resistance to biotrophic and necrotrophic pathogens differently (Lenz *et al.*, 2011). These pleiotropic and antagonistic functions of autophagy, underpinned by a broad array of molecular mechanisms, have been dissected in animals, plants and fungal models. Yet, the potential conservation of these cellular machineries and functions in other eukaryotes remains almost uninvestigated, including in brown algae and oomycetes. So far, studies on autophagy in algae are mostly restricted to few members of unicellular green algae, in the context of responses to stress (e.g. oxidative stress; Bassham & Crespo, 2014), with only a few exceptions looking into stramenopilous algae (Shemi *et al.*, 2015). In brown algae, autophagy was only described in ageing *Ectocarpus* cells (Oliveira & Bisalputra, 1977), and has been linked to differentiation of promeristematic cells in *Cystoseira* (Pellegrini, 1979). The *Ectocarpus* genome contains a core set of autophagy genes (Cock *et al.*, 2010), and transcriptomics suggests the induction of this pathway during abiotic stress (Dittami *et al.*, 2009; Ritter *et al.*, 2014). Autophagy in oomycetes mostly has been investigated in the plant pathogen *Phytophthora*: Chen *et al*. (2017) found that autophagy is a key cellular process in *P. sojae* during its development and in response to starvation. Importantly, *P. infestans* interferes with the autophagy‐related immune responses of potato plants by the release of the effector PexRD54. This effector outcompetes the phagophore host factor Joka2 to bind to an ATG8 protein that is involved in autophagosome formation, thus inactivating the plant autophagy machinery and promoting the progression of the disease (Dagdas *et al.*, 2016; de Figueiredo & Dickman, 2016). Indeed, during *P. infestans* infection, plant ATG8‐containing autophagosomes are diverted to the pathogen interface, suggestive of *P. infestans* subverting the host autophagy for its own benefit (Dagdas *et al.*, 2018).

Thanks to the establishment of a laboratory‐controlled pathosystem, we initiated a detailed transmission electron microscopy (TEM) investigation of the life cycle of *A. ectocarpii* in gametophytes of the giant kelp *M. pyrifera*. This led us to identify a central role of several inducible autophagic responses, both in the host and the pathogen. These responses dictate the outcome of the interaction, namely if the pathogen does or does not complete its development cycle in an infected host cells and releases zoospores. We thus developed protocols to quantify the induction of these responses using vital stains, and tested their sensitivity to a range of autophagy inhibitors, in accordance with accepted experimental standards (Klionsky *et al.*, 2016). We further investigated their functional diversity, as well as the systemic nature of the algal autophagic response.

## Materials and Methods

### Origin and maintenance of *Macrocystis*‐oomycete pathosystems

This study was performed using monoeukaryotic, bacterized cultures of the obligate biotrophic oomycete *Anisolpidium ectocarpii* CCAP 4001/1 (Gachon *et al.*, 2017). Healthy female gametophytes of *Macrocystis pyrifera* CCAP 1323/1 were used as host to propagate *A. ectocarpii*, similar to the protocol described by Strittmatter *et al*. (2013) for *Eurychasma dicksonii*. Culture conditions were settled at 1/2 strength Provasoli medium, at 10°C, under 2–6 µmol photons m^−2^ s^−1^ white light irradiation and 12 h : 12 h, light : dark photoperiod. In order to control bacterial blooms, an antibiotic mix was sometimes supplied (detailed in Coelho *et al.*, 2012). The life stages of *A. ectocarpii* and the experimental set‐ups are illustrated in Supporting Information Figs S1 and S2, respectively.

### Light, epifluorescence and confocal microscopy

Cultures were checked regularly using an Axio‐Observer microscope (Zeiss) coupled with differential interference contrast objectives and recorded on a digital camera (AxioCam HRc; Zeiss). To visualize lipid droplets and their difference in abundance or distribution in *A. ectocarpii*, we used the *in vivo* fluorescent dye Bodipy^®^ (ThermoFisher Scientific, Waltham, MA, USA); the algal material was immersed in 1 µg ml^−1^ solution in sterile seawater, rinsed, mounted with SlowFade^®^ (ThermoFisher Scientific) and observed under the confocal microscope (excitation: 488 nm, emission: 490–535 nm). The fluorescent dyes Monodansylcadaverin (MDC) and Lysotracker red were used to track vacuoles/late autophagosomes and vacuole acidification (e.g. autovacuoles/autolysosome), respectively (Klionsky *et al.*, 2016), sometimes in combination with Calcofluor white (CFW) to stain the pathogen and host cell walls (Gachon *et al.*, 2017). Living algal filaments were incubated for 15 min in 50 µM MDC and 0.01 mg ml^−1^ CFW solution in sterile seawater, rinsed in seawater and observed with a DAPI filter (excitation: 365 nm, beam splitter: 395 nm, emission: long pass 420 nm). To stain acidic vacuoles, samples were dark‐incubated in 1 µM Lysotracker^®^ (ThermoFisher Scientific) solution in fresh seawater at room temperature, then fixed in 4% paraformaldehyde in the fridge overnight, destained in ascending serial dilutions of ethanol and salt‐restored with ascending seawater‐ethanol solutions. Samples were observed with a filter (excitation: 577 nm, emission: 590 nm). Confocal images were acquired with a Zeiss LSM510 microscope.

For time‐course experiments, target *Macrocystis* was co‐incubated with *Macrocystis*–*A. ectocarpii* inoculum for 20 d, in 5‐cm‐diameter Petri dishes with PES (eight cultures inoculated per treatment). A mock‐challenged control was prepared using healthy *M. pyrifera* as an inoculum (Fig. S2). Every day during the first 5 days after inoculation (dai), and then every 5^th^ day until the 20^th^ day, the number of MDC‐positive and MDC‐negative *A. ectocarpii* syncytia and the number of MDC‐positive and MDC‐negative unchallenged host cells were counted in populations of 200–300 host cells. The ratio of MDC‐positive over the total number of *A. ectocarpii* syncytia, and of MDC‐positive over the total number of host cells was determined for every sample. For Lysotracker time series, four *M. pyrifera* cultures were inoculated per treatment. Each culture was harvested every second day for the first 11 d and then after a week. The ratios of (Lysotracker‐positive/total *A. ectocarpii*) and (abortive/Lysotracker positive *A. ectocarpii*) were established by examining 200–500 *M. pyrifera* cells and 200–500 *A. ectocarpii* thalli. For autophagy inhibitor treatments, 17 dai *Anisolpidium* challenged *M. pyrifera* was collected and incubated for 10 d in the following inhibitors: 250 nM and 1 μM Wortmannin (coded WT1 and WT2, respectively); 10 mM 3‐Methyladenine (3‐MA); 0.1 and 0.5 μM Bafilomycin (BM1 and BM2), and 50 and 200 μM Chloroquine (CQ1 and CQ2) (Fig. S2c). Three cultures were inoculated for each inhibitor concentration, and experiments were repeated two times independently. Ratios were calculated as end‐point measurements using the aforementioned dyes.

The variations of these ratios between treatments (time points, inhibitors) were statistically evaluated in R using linear mixed models (LMMs; Bates *et al.*, 2015). Due to the putative physiological heterogeneity of the starting material, the spatial autocorrelation within repetitive samples of same cells or cell populations, and the temporal correlation in our time‐series experiments, the ID of the experiment, the ID of the sampled cell and the sampling day were considered as random effects. When needed, power transformations were performed in the response variables, in order to find the optimum exponent with the slope of a linear regression of log(variance) on log(mean) of the original (observed) data. Homoscedasticity and normality in the final models were confirmed using Levene's and Shapiro's tests and visually after plotting residuals against treatment boxplots and qqplots. Overdispersion was then calculated and < 1. In experiments where normal distributions of the residuals or homogeneity of variance assumptions were not fulfilled, Friedman's ANOVA tests were performed. *Post hoc* Tukey tests with a Bonferroni adjustment were run to determine different statistical groups. Graphs were plotted using R/ggplot2 (Wickham, 2009).

### Electron microscopy

Transmission electron microscopy was done following a high‐pressure fixation pipeline (HPF), using a high‐pressure freezer (Leica Empact 2/RTS, Wetzlar, Germany) combined with an automated freeze substitution system (Leica AFS2/FSP). Unless otherwise stated in the figure legends, the biomass also was chemically fixed (CHF) and processed as described in Murúa *et al.* (2017): the target was immersed in a solution of 2.5% glutaraldehyde, 0.1 M cacodylate buffer (pH 7.4), 0.5% caffeine, 0.1% CaCl_2_ and 3% NaCl in PES for 2–3 d. Further staining steps also involved 1% OsO_4_ and 2% uranyl acetate. After dehydration steps, samples were Spurr‐embedded and polymerized at 60–70°C. Final blocks were sectioned at 90 nm using an ultramicrotome (Leica UC6) and placed on copper grids before being counterstained with lead citrate. Sections were imaged using a JEM‐1400 Plus (Jeol, Akishima, Tokyo, Japan) TEM with an AMT UltraVue camera (Woburn, MA, USA). We used CHF mostly for host plastid observations, as the membrane structures were better fixed and contrasted.

## Results

### Experimental set‐up and terminology used

The infection cycle of *A. ectocarpii* is summarized in Fig. S1. Briefly, the infection starts with a pathogen spore encysting at the surface of the algal host (Fig. S1a), followed by penetration inside a cell. Once infected, host cell plastids and other organelles disintegrate quickly. Originally naked (Fig. S1b,e), the *A. ectocarpii* thallus undergoes several nuclear divisions and ultimately differentiates a walled syncytium inside a progressively digested host cell (Fig. S1c,f). Syncytia are spherical to elliptical in shape; single or multiple infections per host cell are frequent (Fig. S3), but the pathogen never migrates to another cell (Gachon *et al.*, 2017). The syncytia further undergo segmentation and differentiate zoospores, which are released in the medium through a discharge tube (Fig. S1d). Zoospores encyst at the surface of new host cells, leading to the propagation of infection. This cycle takes *c*. 5 d to complete; therefore, in most of our time‐course experiments, several infection cycles were allowed to occur.

The inoculation procedure used throughout this study is illustrated in Fig. S2(a): originally healthy algal tufts (the ‘target culture’) are ‘challenged’ with an infected inoculum that releases pathogen spores. In the ‘challenged target culture’ (Fig. S2b), we distinguish: ‘infected cells’ (which contain one or more intracellular pathogen thalli), ‘challenged host cells’ (which have at least one encysted spore at their surface, but do not necessarily contain an intracellular thallus), and ‘unchallenged host cells’ (which do not show any evidence of infection, nor of challenge by the pathogen). As a mock control (the ‘mock‐challenged control’), a target alga was co‐incubated with a healthy tuft in lieu of the inoculum. It is important to note that in time‐course experiments, the ‘target’ algal tufts are continuously challenged by spores released by the inoculum and the maturing sporangia of the target (i.e. reinfection; Fig. S2c).

### Inducible autophagy in *A. ectocarpii* in relation to its lipid content and zoosporogenesis

**Figure 1:**
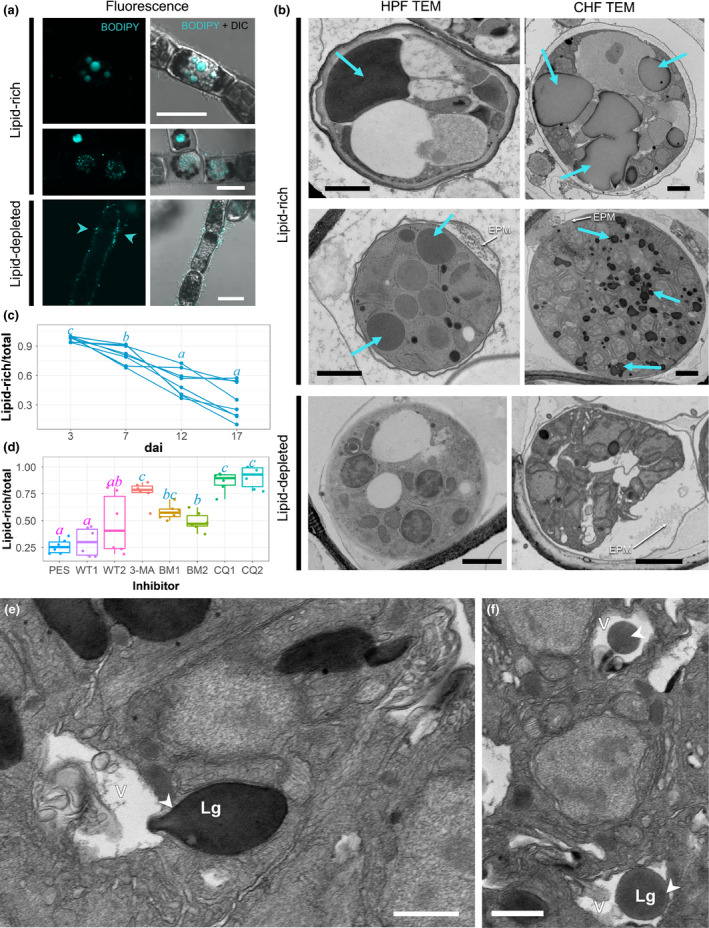
The developmental plasticity of *Anisolpidium ectocarpii* over time relates to lipid mobilization and depends on the induction of lipophagy. (a) BODIPY^®^ staining highlights that *A. ectocarpii* thalli exhibit a varying abundance lipid globules; the top left panel shows BODIPY‐positive thalli (hereafter called lipid‐rich thalli) with with globules of varying sizes. The bottom left panel shows a BODIPY‐negative *A. ectocarpii* thallus (hereafter called lipid‐depleted; note that epiphytic bacteria are stained, arrowheads). Bars, 12 μm. (b) The same stages can be recognized in transmission electron microscopy (TEM), after high pressure (HPF) and chemical (CHF) fixation. Top panel: thalli with a small number (1 or 2) of prominent lipid globules are usually poorly fixed, but note that no thallus shrinkage (e.g. appearance of periplasmic space) is visible. Middle panel: several lipid globules (Lg) of micrometric diameter can be seen; note at this stage, the *A. ectocarpii* thallus has reduced evaginations of plasma membrane (EPM), no periplasmic space, and almost no visible vacuolization. Bottom panel: lipid‐depleted *A. ectocarpii* syncytia (Sy) typically exhibit vacuolization and shrinkage, leading to a prominent periplasmic space. The latter commonly contain prominent EPMs, which are observed in CHF and HPF samples. See Supporting Information Fig. S3 for an example of a vacuolated syncytium with prominent EPMs under HPF, and close‐ups for further ultrastructural details. Cyan arrowheads: lipid globules. Bars, 12 μm (fluorescence pictures), 500 nm (TEM images). (c) The proportion of *A. ectocarpii* thalli containing lipid globules decreases over the time course of algal infection. Each curve corresponds to an independent replicate; the letters above every sampling day designate the statistically significant differences between time‐points (Tukey test after linear mixed model), where a < b < c and *P* < 0.05. (d) The depletion of lipids in *A. ectocarpii* is compromised by exposure to inhibitors of autophagy. The proportion of lipid‐rich thalli in each treatment (calculated as in b) is plotted after a 10‐d incubation. PES, Control (Provasoli‐Enriched Seawater only); WT1 and WT2, 250 nM and 1 μM Wortmannin, respectively; 3‐MA, 10 mM 3‐Methyladenine; BM1 and BM2, 0.1 and 0.5 μM Bafilomycin, respectively; CQ1 and CQ2, 50 and 200 μM Chloroquine, respectively. Horizontal lines represent median values and scatterplots all of the independent measurements per treatment. Letters above each box designate the statistically significant groups (Tukey test after linear mixed model), where a < b < c and *P* < 0.05. (e–f) Lipid depletion in *A. ectocarpii* syncytia (CHF) appears mediated by both piecemeal autophagy (e) and microautophagy (f). Arrowhead in (e): sequestration of a small piece of the targeted lipid globule (Lg) by the vacuole (v). Arrowhead in (f): incorporation of the entire lipid globule (Lg) in the vacuole (v). Bars, 500 nm.

The vital dye BODIPY^®^ allowed us to distinguish several patterns of lipid abundance, droplet size and spatial distribution in *A. ectocarpii* (Fig. 1a), which we could associate with HPF and CHF TEM observations (Fig. 1b). First, we observed lipid‐rich *A. ectocarpii* thalli with lipid globules of variable size: typically, young *A. ectocarpii* thalli have a small number of rather large (> 1 μm diameter) globules, whereas multinucleate syncytia have multiple smaller (< 1 μm diameter) globules; these lipid‐rich thalli contrasted with lipid‐depleted thalli in which no intracellular BODIPY signal was detected (Fig. 1a). Over a 17‐d time course, the proportion of lipid‐rich *A. ectocarpii* thalli steadily decreased, illustrating a nutritional shift of the *A. ectocarpii* populations from lipid‐rich at the beginning of the infection to mostly lipid‐depleted after several infection cycles (Fig. 1c; *P* < 0.05). A subsequent 10‐d treatment of *A. ectocarpii* with autophagy inhibitors led to a significant accumulation of lipid‐rich thalli with 3‐MA, bafilomycin and chloroquine (Fig. 1d; *P* < 0.05; see representative images in Fig. S4). This suggests that autophagy is necessary for lipid consumption. In support of this interpretation, lipid‐vacuolar junctions often were observed in *A. ectocarpii* thalli of untreated control cultures (only PES): whereas teardrop invaginations of lipid droplets inside vacuoles – suggestive of piecemeal digestion – were sometimes observed (Fig. 1e), the engulfment of entire globules was more common (Fig. 1f).

**Figure 2:**
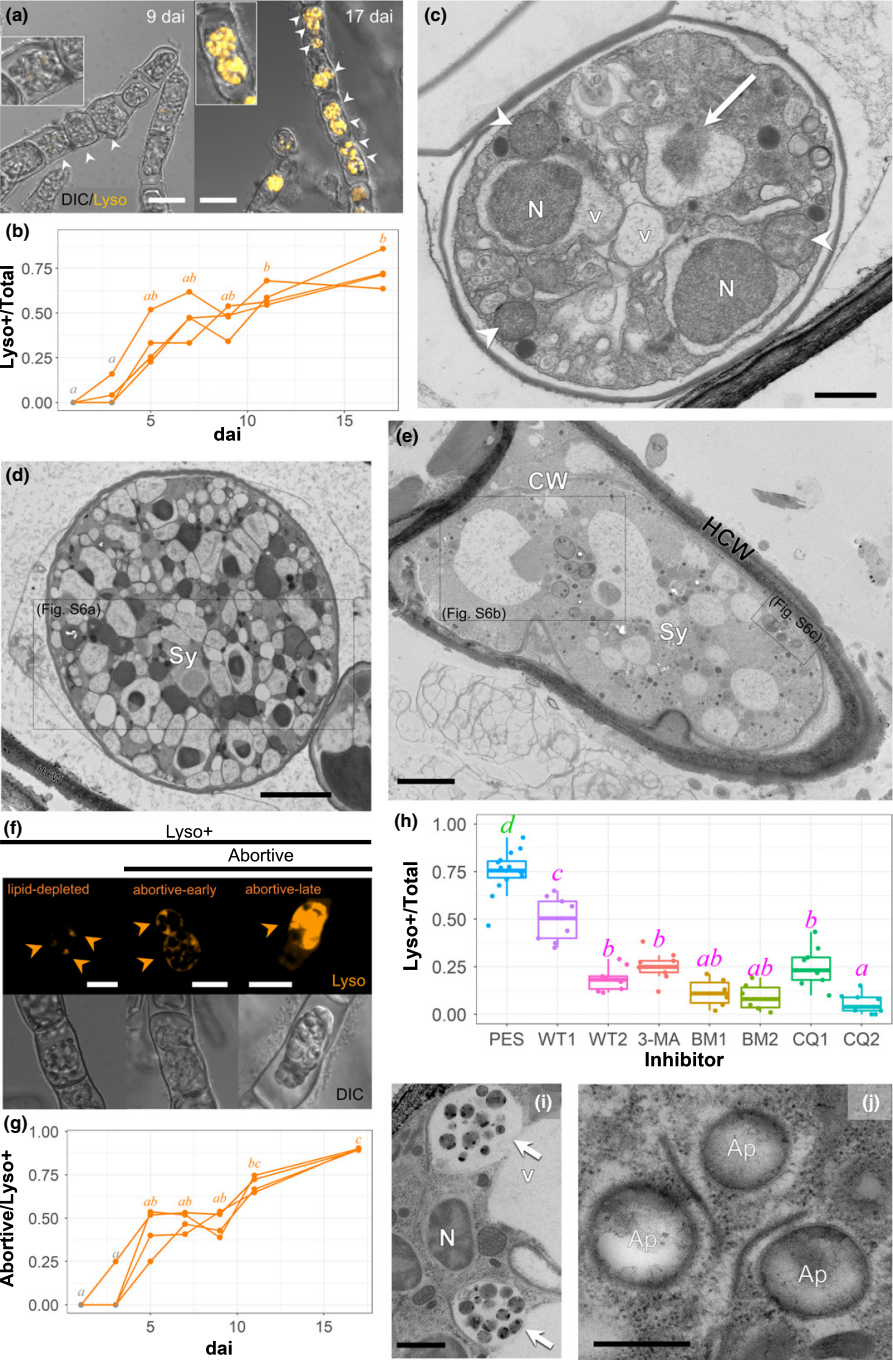
Induction of vacuolar acidification and thallus abortion in *Anisolpidium ectocarpii* depends on autophagic processes. (a) *Macrocystis pyrifera* filament with Lysotracker‐negative (left) and Lysotracker‐positive (right) *A. ectocarpii* thalli, 9 and 17 day after inoculation (dai), respectively. Arrowheads: *Anisolpidum* thalli (magnified in insets)*.* DIC/Lysotracker overlay confocal images; bar, 15 μm. (b) Accumulation of Lysotracker‐positive thalli in the *A. ectocarpii* population over time. The y‐axis represents the ratio of Lysotracker‐positive *A. ectocarpii* thalli over the total number of pathogen thalli examined. Individual time courses are shown for four separate inoculations and are directly comparable to the Monodansylcadaverine (MDC) data presented in Fig. S5. Time‐points where Lysotracker positive *A. ectocarpii* were not found are designated as grey dots. Letters on every sampling day designate the statistically significant differences between time points (Friedman tests), where a < b (*P* < 0.05), after Tukey test for multiple comparisons. (c) Transmission electron microscopy of chemically‐fixed samples (CHF TEM) micrograph of an early walled *A. ectocarpii* syncytium with intact organelles (e.g. mitochondrion, arrowheads), limited periplasmic space and digestive vacuoles (v) containing nuclei (N). The white arrow points to a partially digested nucleus. Bar, 500 nm. (d) Highly vacuolated, abortive *A. ectocarpii* syncytium (Sy) at an early stage. Note the limited periplasmic space seen under TEM of samples fixed by high‐pressure freezing (HPF TEM), and absence of EPMs. HCW, host cell wall. Bar, 2 μm. (e) Late abortive *A. ectocarpii* syncytium (Sy) under HPF TEM: no clear organelle remains, and digestive vacuoles are abundant. CW, cell wall; HCW, host cell wall; P, papilla (see details in Fig. 4). Bar, 2 μm. (f) Lysotracker staining (arrowheads) enables discrimination between ‘lipid‐depleted’ and ‘abortive’ *A. ectocarpii* autophagic thalli, as defined in Figs 1(a) and 2(c–e), respectively. In the lipid‐depleted image, targeted spore degeneration corresponds to punctate Lysotracker staining (orange arrowheads). By contrast, abortive thalli exhibit Lysotracker positive vacuoles in their entire volume, which become increasingly stained as degeneration progresses. Bar, 8 μm. (g) Accumulation of abortive thalli in the population of autophagic *A. ectocarpii* thalli over time. The y‐axis represents the ratio of abortive *A. ectocarpii* thalli over the total number of Lysotracker‐positive pathogen thalli examined. Individual time courses are shown for four separate inoculations. Time‐points where lipid‐depleted, but no abortive *A. ectocarpii* were found are designated as grey dots. Letter on bars designate the statistical differences after the multiple comparisons between groups (Friedman tests), where a < b < c (*P* < 0.05) after Tukey's multiple comparisons. (h) Lysotracker‐positive ratio in *A. ectocarpii* after 10 d exposure to different autophagy inhibitors. PES, Control without inhibitor (Provasoli‐enriched seawater medium); WT1 and WT2, 250 nM and 1 μM Wortmannin; 3‐MA, 10 mM 3‐Methyladenine; BM1 and BM2, 0.1 and 0.5 μM Bafilomycin. CQ1 and CQ2, 50 and 200 μM Chloroquine. Letters on every sampling day designate the statistically significant differences between time‐points (Linear mixed model), where a < b < c (*P* < 0.05) after Tukey's multiple comparisons. (i–j) under the effect of autophagy inhibitors (i = CQ2 and j = BM2), vacuolated *A. ectocarpii* syncytia with (i) debris and (j) double membrane autophagosomes very common in HPF images. Arrow: undigested debris inside central vacuole (v); Ap, autophagosomes; N, nucleus. Bars: 1 μm (i) and 100 nm (j).

Examination of high‐pressure and chemically fixed infected cultures using TEM allowed us to correlate ultrastructural features with these lipid content variations (Figs 1b, S3). Lipid‐rich thalli are characterized by prominent and abundant lipid globules, small empty vacuoles (if present) and several nuclei with condensed heterochromatin (Figs 1a, S3c,h). Their periplasmic space is reduced; membrane evaginations in the apoplast are normally present but are reduced in size (Fig. 1b, middle panel; Fig. S3f,g). By contrast, lipid‐depleted *A. ectocarpii* syncytia appear shrunk inside their cell wall, often displaying a prominent periplasmic space filled by conspicuous evaginations of the plasma membrane (hereafter EPMs) (Fig. 1a, bottom panel; Fig. S3i,j). Additionally, lipid‐depleted syncytia often show degeneration of some of their spore initials (arrowhead in Fig. S3j, also shown in Fig. 2f), whilst other spores in the sporangium appear to develop normally; this selective degeneration of spore initials was observed regardless of the thallus size, suggesting that it regulates the number of spores produced by each *A. ectocarpii* thallus depending on the available resources, as described by Gachon *et al*. (2017). In conclusion, we find that over several infection cycles, the proportion of lipid‐rich *A. ectocarpii* thalli decreases overall in the pathogen population; that lipid mobilisation is dependent on autophagic processes; and that lipid depletion correlates with the targeted degeneration of some spore initials, thallus shrinkage and sometimes differentiation of EPMs in the periplasmic space.

### Inducible autophagy in *A. ectocarpii*: a temporal shift from lipid mobilization to thallus abortion

We used Lysotracker (Fig. 2a) and MDC (Fig. S5) to follow the appearance of acidic vesicles in *A. ectocarpii* thalli over time. The first *A. ectocarpii* thalli were recorded in target cultures at 1 dai. The first Lysotracker‐positive thalli were observed at 3–5 dai, depending on the replicate observed (Fig. 2b), and the first MDC‐positive thalli were observed from 3 dai onwards (Fig. S5d). The proportion of MDC‐positive thalli in the *A. ectocarpii* population increased significantly from 3 dai onwards, reaching values ranging from 70% to close to 100% at 5 dai (*P* < 0.05; Fig. S5d). The response was a bit slower, yet still significant with Lysotracker: roughly three quarters of *A. ectocarpii* thalli were Lysotracker‐positive at 17 dai (Fig. 2b).

Strikingly, we observed the progressive appearance of highly vacuolated *A. ectocarpii* thalli that appeared to degenerate entirely from 7 dai onwards (Figs 2c–e, S6a–c), in addition to the lipid‐depleted *A. ectocarpii* syncytia described in Fig. 1(a,b). These abortive syncytia can be of any size and contain a variable lipid content; they have limited periplasmic space, inconspicuous EPMs, may contain intact organelles (e.g. mitochondria on Fig. 2c) and contain several digestive vacuoles. Abortive syncitia also are present in dead host cells, as judged by the absence of recognisable organelles (e.g. Fig. 2d,e). Remarkably, the *Anisolpidium* organelles that first undergo digestion in abortive syncytia strongly resemble whole nuclei engulfed in vacuoles, whereas intact nuclei are missing from the pathogen cytosol (Figs 2c,d, S6a). The condensed heterochromatin of these nuclei undergoing digestion is most recognizable under HPF (Figs 2d, S6b; cf. a nonautophagic thallus fixed with the same method in Fig. S3b,c). At a later stage, abortive *A. ectocarpii* syncytia do not contain any recognizable organelles, apart from many digestive vesicles and vacuoles (Figs 2e, S6b,c). Some of the vesicles appear to be delimited by a double membrane and may thus correspond to phagophores (Fig. S6c).

As shown on Fig. 2(f), Lysotracker enabled us to discriminate staining patterns that correlated with the lipid‐depleted and abortive vacuolated phenotypes observed under TEM: lipid‐depleted *A. ectocarpii*, with one or a few degenerative spore initials, displayed punctate staining (cf. Fig. S3j for an equivalent TEM image), whereas early abortive syncytia had punctate Lysotracker‐positive areas and Lysotracker‐negative vacuoles (cf. Fig. 2c,d), and late abortive *A. ectocarpii* had nearly all their biovolume stained (cf. Fig. 2e). We were thus able to show that amongst Lysotracker‐positive thalli, the proportion of abortive *A. ectocarpii* was initially null, and steadily increased to nearly 100% at 17 dai (*P* < 0.05; Fig. 2g).

Seventeen‐day‐old infected cultures were further incubated for 10 d with autophagy inhibitors as described in Fig. S2(c), and checked for Lysotracker (Figs 2h, S7) or MDC signal (Figs S5e,f, S8), as well as ultrastructural modifications using HPF TEM (Figs 2i,j, S6d–r). Whereas in the control group without inhibitor (PES medium only) the proportion of Lysotracker‐positive *A. ectocarpii* thalli remained high at 27 dai, these ratios were diminished and sometimes almost abolished with all the autophagy inhibitors used (*P* < 0.05; Fig. 2h). A similar trend was observed with MDC, with ratios significantly decreased to < 50% of the *A. ectocarpii* population after 3‐MA and chloroquine treatment (*P* < 0.05; Fig. S5e). Regular observations during the 10‐d treatment showed that the effect of these inhibitors on MDC staining increases over time (Fig. S8). Autophagy inhibitors also had an impact on A*. ectocarpii* ultrastructure (Figs 2i–j, S6d–q): (1) a significant accumulation of lipids was observed in almost all of the treatments (Fig. S6d–q), confirming our quantifications using BODIPY (Fig. 1d); (2) highly vacuolated thalli were very abundant with all inhibitor treatments, especially with Wortmannin and 3‐MA (Fig. S6d–i); (3) nuclei inside vacuoles were virtually absent, except with Wortmannin (Fig. S6d,e); (4) with bafilomycin and sometimes with chloroquine, double‐membrane structures resembling autophagosomes were found (Fig. S6k,m,q), suggesting a disruption of their fusion with the main vacuole; (5) additionally, treatment with chloroquine led to the accumulation of vacuoles with debris (Figs 2i,j, S6n–p). In conclusion, the combination of TEM with MDC and Lysotracker staining reveals quantitative, sequential shifts in the physiological status of the *A. ectocarpii* population over time. Initially, lipid‐rich thalli develop successfully, followed by the appearance of vacuolated and lipid‐depleted thalli that undergo targeted spore degeneration, yet successfully complete their development cycle. At later time points, the *A. ectocarpii* population becomes dominated by wholly abortive thalli digesting all of their nuclei irrespective of lipid content, which stops the pathogen development cycle. When applied on such cultures dominated by abortive thalli, all autophagy inhibitors (to some extent at least): restore the accumulation of lipid globules, lead to high vacuolization but limited digestion of cellular debris, abolish the loss of nuclei, and (4) lead to the accumulation of autophagosomes.

### Local algal defences and pathogen elimination via xenophagy

**Figure 3:**
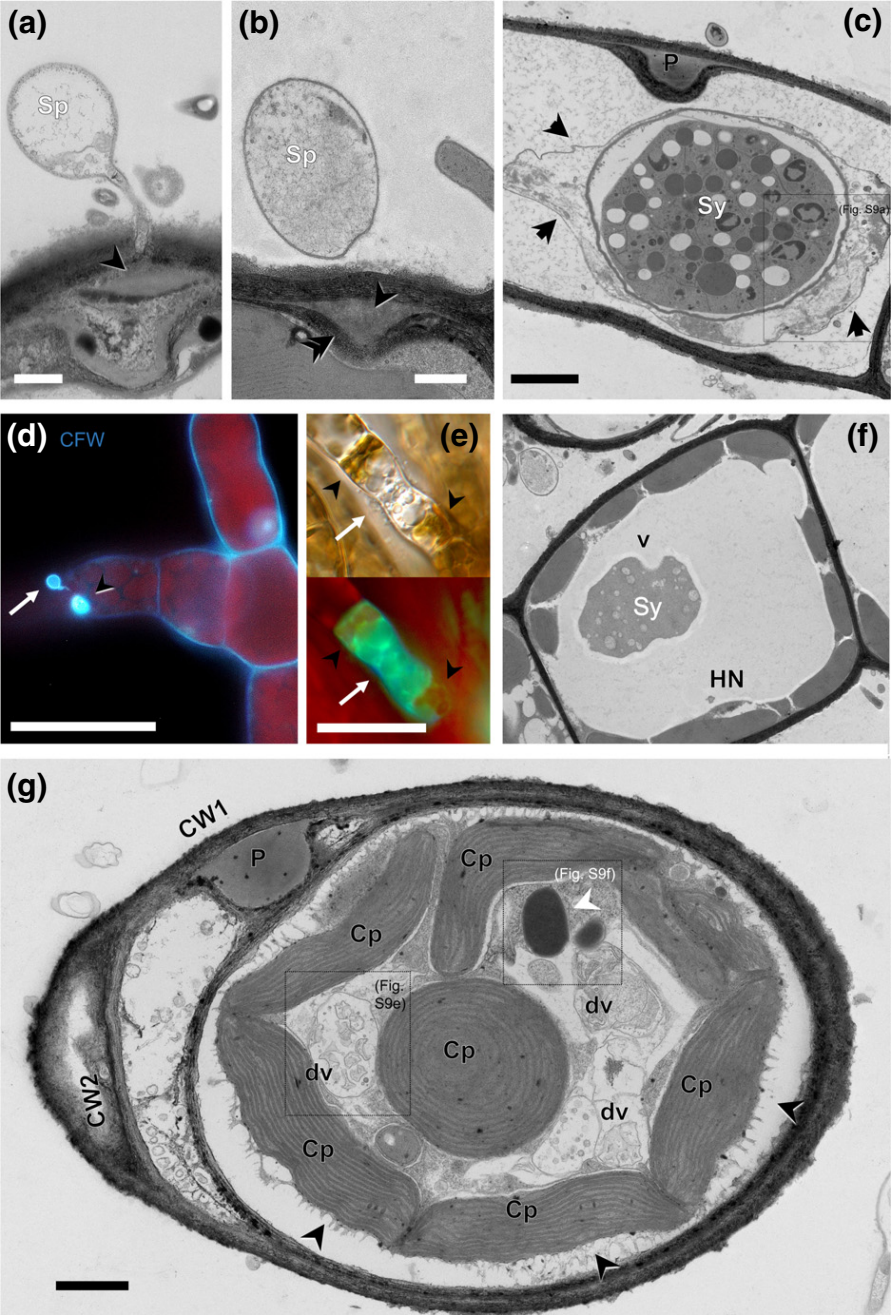
Local cellular modifications in *Macrocystis pyrifera* challenged with *Anisolpidium ectocarpii*, and its intracellular elimination via xenophagy. (a–c) Formation of a papilla by the host cell at the site of pathogen penetration, revealed by transmission electron microscopy of samples fixed by high‐pressure freezing (HPF TEM). (a) Localized deposition of amorphous material (arrowhead) underneath a germinated spore (Sp). Bar, 500 nm; (b) subsequent deposition of fibrillary material (white arrowhead) underneath the amorphous plug (arrowhead). Bar, 500 nm; (c) fully developed bi‐layered papilla (P). In addition, note the ectopic deposition of a thin fibrillary layer on the collapsed plasmalemma (arrowheads) of the host cell, which is successfully infected by *A. ectocarpii* (Sy). Bar, 2 μm. (For ultrastructural details, see Fig. S9a,b) (d) A papilla (arrowhead), as seen under epifluorescence microscopy, after *A. ectocarpii* encystment and penetration attempt (arrow). CFW, Calcofluor white. Bar, 18 μm. (e) Both *M. pyrifera* and the intracellular *A. ectocarpii* with positive Monodansylcadaverine (MDC)‐labelling. Arrow: pathogen; Arrowheads: host cellular content showing MDC‐positive labelling. Bar, 15 μm. (f) HPF micrograph of a structurally undisrupted *M. pyrifera* with a conspicuous digestive vacuole (dv) containing a vacuolized, highly degraded pathogen thallus (Sy), suggestive of xenophagy of a young, unwalled *A. ectocarpii* syncytium. Such digestive vacuoles are never observed mock‐challenged *M. pyrifera* (refer to Fig. S10 for representative examples of mock‐challenged cells in HFP and CHF). HN, Host nucleus. Bar, 2 μm. (g) CHF image. In a challenged host cell (as evidenced by the formation a papilla, P), successful defence against *A. ectocarpii* is marked by the ectopic deposition of a second fibrillary cell wall layer surrounding the entire host cell (CW2). The host organelles retreat inwards, with digestive vacuoles (dv) containing cellular debris. The white arrowhead points to an object resembling a lipid globule, never observed in healthy *M. pyrifera*, and which we therefore interpret as a cellular debris originating from *A. ectocarpii* and undergoing digestion. CW1, original host cell wall; Cp, chloroplast; Bar, 1 μm. For ultrastructural details, see Fig. S9(d–f).

In addition to the changes described above for the pathogen, we also observed ultrastructural changes in challenged *M. pyrifera* cells. After a penetration attempt by *A. ectocarpii*, a discrete plug of amorphous material is rapidly deposited just underneath the host cell wall (Fig. 3a), immediately followed by a secretion of a second layer of fibrillar material (Fig. 3b). This process results in the formation of a bilayered papilla (labelled ‘P’ in Fig. 3c) and is accompanied by an ectopic deposition of fibrillar material along the entire host plasma membrane (arrowheads in Fig. 3c; see detail on Fig. S9a,b). If this defence process fails – as judged by the successful development of an *A. ectocarpii* syncytium inside the host – the resulting layer looks disrupted, yet thicker than a collapsed plasma membrane would (Fig. 3c, see detail on Fig. S9a,b). However, when this response is successfully deployed, a new cell wall is differentiated underneath the papilla (noted CW2 on Figs 2g, S9c). Papillae and the secondary cell wall are stained with CFW and thus are easily identifiable under epifluorescence and confocal microscopy (Fig. 3d).

Interestingly, some algal cells challenged by *A. ectocarpii*, as judged by the presence of a papilla, a secondary cell wall and/or an early *A. ectocarpii* syncytium, show additional structures absent in a mock‐challenged *M. pyrifera* control, and all highly suggestive of recovery from infection (Figs 3e–g, S9c,d; see Fig. S10 for a comparison with mock‐challenged host cells). Under TEM, such recovered cells also exhibit plastids that appear smaller than those of mock‐challenged *M. pyrifera* (no quantification was attempted with TEM because this stage was too uncommon). MDC staining also revealed cells in which both the host and pathogen were stained (Fig. 3e), that we were able to correlate with TEM images of unwalled, vacuolized and highly degraded *A. ectocarpii* in the central vacuole of *M. pyrifera* cells (Fig. 3f). Some algal cells also visibly challenged by *A. ectocarpii* because of a well‐developed papilla and secondary cell wall visible in TEM exhibited several uncommon structures in a healthy *M. pyrifera* culture (Fig. 3g): large (*c*. 1.5 μm) (Fig. S9d), irregular vacuoles filled with either unrecognizable cellular debris (Fig. S9e) or cellular elements of unambiguous *A. ectocarpii* origin (e.g. lipid globules in Figs 3g, S9f; cf. *A. ectocarpii* thalli in Fig. 1b). Altogether, our observations show that challenged algal cells mount local defences that may or may not be successful in stopping the pathogen: this local response starts with the deposition of a papilla, potentially followed by ectopic cell wall thickening and plastid division; it may culminate with successful recovery and digestion of the oomycete intruder by xenophagy.

### A systemic algal response is mediated by autophagy

**Figure 4:**
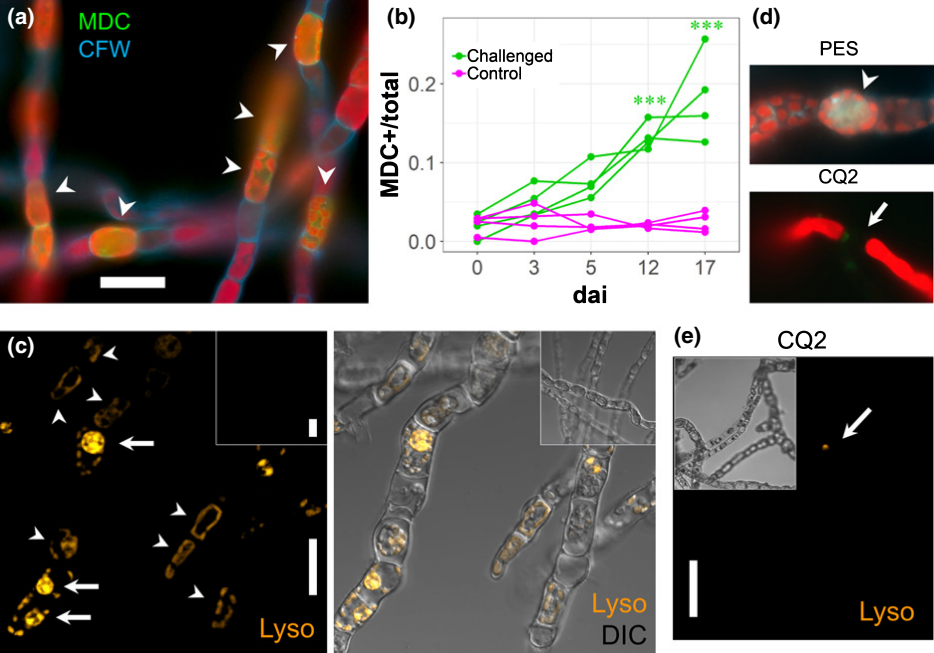
Autophagy is an inducible response in unchallenged algal cells. (a, b) Unchallenged *Macrocystis pyrifera* cells in an *Anisolpidium‐*challenged culture become autophagic over time. (a) Double CFW and Monodansylcadaverine (MDC) staining of an infected culture, 17 day after inoculation (dai). Unchallenged *M. pyrifera* cells (as defined in Fig. S2) are MDC‐positive (arrowheads). Bar, 18 μm. (b) The proportion of MDC‐positive unchallenged host cells increases steadily in an *Anisolpidium*‐challenged culture during a 17‐d infection time‐course, whereas it remains consistently low in a mock‐challenged control (*n* = 4). The distinction between challenged and unchallenged cells was made using CFW, which quantitatively stains host papillae and encysted *Anisolpidium* spores. Asterisks designate statistically significant differences between time‐points (Tukey multiple comparisons after Friedman test, *P* < 0.05). (c) Lysotracker staining of infected *M. pyrifera* cultures 11 dai (main panels: Lysotracker and Lysotracker/DIC overlay of confocal channels). Arrows point to *A. ectocarpii* autophagic thalli. Arrowheads point to Lysotracker‐positive, yet unchallenged *M. pyrifera* cells. A mock‐challenged *M. pyrifera* control (insets) remains consistently Lysotracker‐negative over the same time course. Bar, 20 µm. (d, e) MDC (d) and Lysotracker staining (e) of infected *M. pyrifera* cultures, following treatment with Chloroquine (CQ2). See Figs S11(b) and S12 for MDC and Lysotracker staining (respectively) following all inhibitor treatments and their effect on the staining of unchallenged host cells.

Time‐course experiments showed that unchallenged host cells of a target *M. pyrifera* culture challenged with *A. ectocarpii* exhibited increasing MDC labelling over time (Fig. 4a,b): although only 3% of the host cells were MDC‐positive at 0 dai (mostly apical or damaged cells; Fig. S11a), on average 18% of unchallenged algal cells were MDC‐positive at 20 dai (*P* < 0.05; Fig. 4b). By contrast, the proportion of MDC‐positive cells in a mock‐challenged *M. pyrifera* culture remained low (*c*. 3% on average) constant throughout the experiment.

Confocal imaging of Lysotracker‐stained challenged target cultures also supported an onset of vacuolar acidification in unchallenged host cells, compared to mock‐challenged *M. pyrifera* (Fig. 4c; note that no quantification was attempted with this time‐consuming method). A subsequent incubation with autophagy inhibitors resulted in the almost complete loss of both MDC and Lysotracker signals in unchallenged cells, whereas the untreated control with only PES medium remained stained (Figs 4d,e; S11b, S12).

In TEM, unchallenged host cells within *A. ectocarpii*‐challenged cultures were recognized by the absence of *A. ectocarpii* structures and of papillae; such unchallenged cells are often adjacent/close to an infected cell (Fig. S13a), and exhibit several hallmarks of autophagy consistent with our Lysotracker and MDC observations. This includes formation of multiple vesicles underneath the plasmalemma, enlarged Golgi bodies, digestive vacuoles and dividing plastids (Fig. S13a–d).

**Figure 5:**
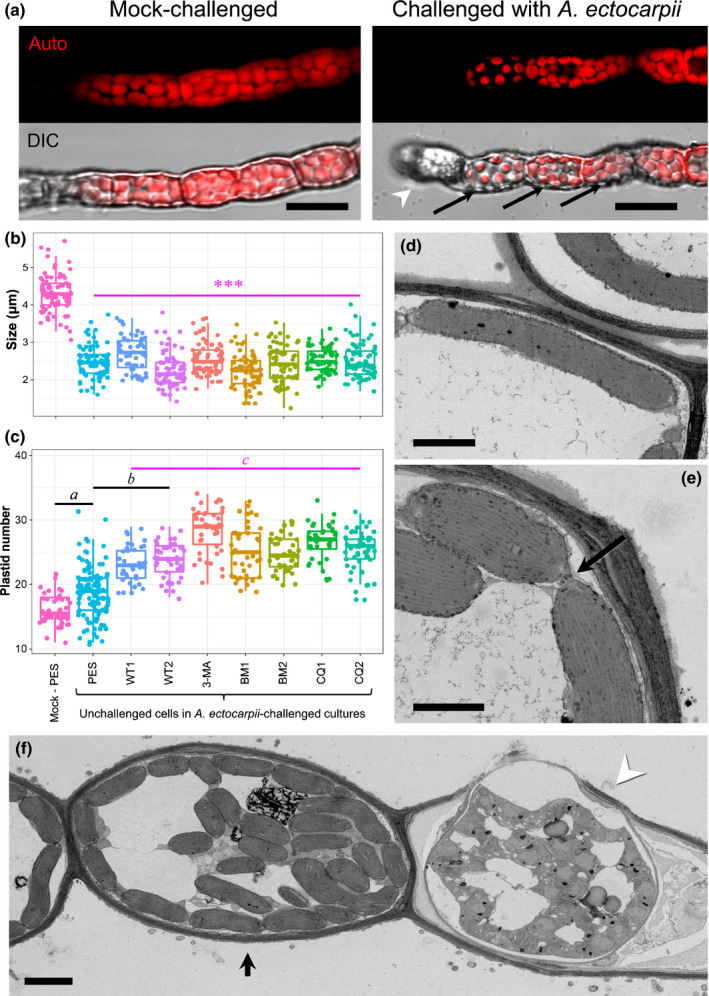
Systemic plastid recycling in infected *Macrocystis pyrifera* is sensitive to autophagy inhibitors. (a) Confocal observation of plastid autofluorescence (top) and autofluorescence/DIC (bottom) of mock‐challenged (left) and *Anisolpidium*‐challenged (right) *M. pyrifera* filaments. Plastids appear reduced in size in unchallenged host cells, especially those closest to the infected cell visible on the left. Auto, Chlorophyll autofluorescence. Arrowhead: infected cell. Arrows: unchallenged cells. Bars, 12 μm. (b, c) Comparison of plastid size (b) and number per *M. pyrifera* cell (c) between a mock‐challenged culture and *Anisolpidium*‐challenged cultures, 10 d post‐treatment with different autophagy inhibitors, using confocal (z‐stacked) microscopy. Mock PES, mock‐challenged *M. pyrifera* in only Provasoli‐enriched seawater. Unchallenged cells in a challenged culture: PES, Control for inhibitor (Provasoli‐enriched seawater only); WT1 and WT2, 250 nM and 1 μM Wortmannin; 3‐MA, 10 mM 3‐Methyladenine; BM1 and BM2, 0.1 and 0.5 μM Bafilomycin; CQ1 and CQ2, 50 and 200 μM Chloroquine. Statistical differences after Linear mixed model expressed with asterisks (***, *P* < 0.001) or superscript letters where a < b < c, with *P* < 0.001 (Tukey's test for multiple comparisons). (d, e) Transmission electron microscopy of chemically‐fixed samples (CHF TEM) reveals that small‐sized plastids correlate with frequent division in unchallenged cells and arrow dividing plastid (e), a process much less common in mock‐challenged *M. pyrifera*, where plastids are normally elongated (d). See Fig. S14 (c–f) for details. Bars, 2 μm. (f) Representative CHF TEM image illustrating the accumulation of small plastids in a *M. pyrifera* cell after treatment with 250 nM Wortmannin (WT1). The accumulation of small‐sized plastids in unchallenged cells following the application of other autophagy inhibitors is shown on Fig. S14(g) and black arrow unchallenged cell neighbouring an infected cell (f). Arrowhead: infected cell. Bar, 2 μm.

We quantified the variations of plastid size and number in unchallenged cells compared to mock‐infected cultures with confocal (z‐stack) and TEM microscopy (Figs 5, S14a–f). With both approaches, we found that the average plastid length was roughly halved in unchallenged cells of cultures challenged with *A. ectocarpii*, compared to a mock‐challenged *M. pyrifera* control (*P* < 0.001; Figs 5a,b, S14a); however, plastids were not significantly more abundant in these unchallenged cells compared to the mock‐challenged control (*P* < 0.001; Figs 5c, S14b). Following treatment with autophagy inhibitors, the plastids of unchallenged cells remained of similar size as in the control treatment without inhibitor (and therefore smaller than the mock‐challenged control), but their number was significantly higher (*P* < 0.05; Figs 5b,c, S14a,b). TEM observations revealed frequent figures of plastid divisions in unchallenged cells (Figs 5d,e, S14c–f); numerous smaller plastids accumulate in unchallenged cells following treatment with autophagy inhibitors (Figs 5f, S14g). Despite limitations resulting from the difficulty of fixing samples, some hallmarks of plastid autophagy were observed in unchallenged *M. pyrifera* cells (Fig. S15): this includes the engulfment of entire small chloroplasts reminiscent of microautophagy as well as several plastid–vacuolar junctions in challenged cultures.

Overall, our data show that the smaller plastid size in unchallenged cells is attributable to the systemic induction of plastid division during infection; yet, plastid number in unchallenged cells remains constant unless autophagy inhibitors are applied. We conclude that a systemic response to infection is mounted in unchallenged cells, and hypothesize that inducible chlorophagy is a component of this systemic response.

## Discussion

**Figure 6:**
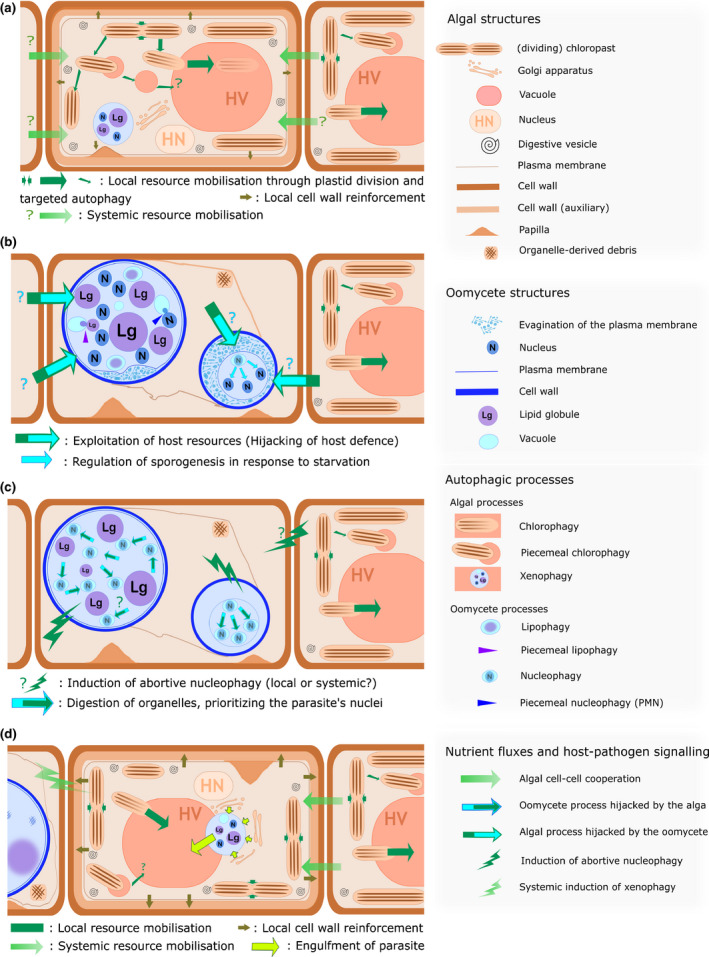
Induction and mutual hijacking of autophagy in brown algal*–*oomycetes interaction: a working model. (a) Induction of autophagy in the host in response to the oomycete. The division and controlled digestion of organelles allows for the onset of defences, such as quick cell wall reinforcements initially localized (papilla). The autophagic structures and chloroplast divisions observed both in challenged and unchallenged cells point to a local and systemic response for resource mobilization. HN, host nucleus; HV, host vacuole. (b) First outcome: the infected host cell perishes and the oomycete completes its life cycle. The oomycete uses nutrients from the host to develop and accumulate lipid globules (Lg) as reserves, which are used by lipophagy/piecemeal lipophagy. When algal resources become exhausted (e.g. in a second, later infection of the same host cell), Lgs are absent and the parasite responds to starvation putatively by inducing a bigger evagination of the plasma membrane (EPM). Some spore initials might be sacrificed, allowing a few to be rescued. It remains open whether the local and systemic nutrient recycling incurred by the algal autophagy is hijacked by the pathogen for its own trophic benefit. (c) Second outcome: both the host cell and the parasitic syncytium die off. Irrespective of its trophic status (as judged by the presence or absence of Lgs), the oomycete becomes fully autophagic and prioritizes digestion of all of its nuclei (nucleophagy). It is assumed that this suicide is triggered by the host cell as a last line of defence before its imminent death, forcing it down the path of intruder cell suicide and avoiding sporogenesis of the parasite; whether or not systemic signalling contributes to this response remains to be established. (d) Third outcome: the host survives and the oomycete is neutralized. Once fully induced, the algal autophagic response described in (a) culminates with the complete synthesis of a new wall all around the cell. Xenophagy directly kills the invader. The signals and cell–cell communication events underpinning the host's systemic response remain to be investigated.

Our work reveals that the induction of autophagic processes is central in deciding the outcome of the interaction between the oomycete *Anisolpidium ectocarpii* and its brown algal host *Macrocystis pyrifera*; the sequential induction of diverse host and pathogen autophagic mechanisms dictates the successful development or elimination of the pathogen, through mechanisms equally complex to those described in animal, yeast and plant systems. Autophagy also is the first cellular process ever linked to a systemic response to disease in a multicellular alga. In the following sections, we fit our observations into a working model that highlights the possible functions and physiological significance of each process (Fig. 6).

### Inducible, systemic host autophagy may enable energy mobilisation towards defences

Our data on the size and number of plastids establish that extensive chloroplast digestion is induced during infection systemically (in unchallenged cells). The smaller size of plastids in recovered cells (e.g. Fig. 3f) suggests that this also might occur locally in infected cells, irrespective of whether they overcome infection or not. Quantifications in unchallenged cells show that the number of plastids remains constant despite recurrent divisions; this number increases after treatment with all autophagy inhibitors tested, which demonstrates that plastids are being degraded via (an) autophagic process(es) following their division. This systemic response implies the existence of cell‐to‐cell communication despite the localized nature of the infection.

Infected cells induce costly defence mechanisms, such as the deposition of a papilla and secondary cell wall, a response conserved with other pathogens such as the oomycete *Eurychasma dicksonii* (Tsirigoti *et al.*, 2015). The scarcity of live infected cells in our transmission electron microscopy (TEM) observations suggests that this stage is very transient and therefore, that these local responses are deployed very quickly. We thus hypothesize that the autophagic recycling of organelles mediates the rapid mobilization of energy required for mounting local defences (Fig. 6a). This hypothesis fits the general rule that plants, animal and fungal models trigger autophagy after starvation as a mean to remobilize nutrients (Rose *et al.*, 2006; Melendez & Neufeld, 2008; Shpilka *et al.*, 2015). Autophagy‐related genes also are induced during nitrogen depravation in the diatom *Phaeodactylum tricornutum*, the biological model most closely related to brown algae in which this response has been investigated (Alipanah *et al.*, 2015). Indirect evidence suggests that cell‐to‐cell communication may underpin intercellular cooperation, notably a potential transfer of nutrients from unchallenged to infected cells. Indeed, such a cell‐to‐cell nutritional cooperation would predictably lead to a progressive depletion of resources across entire algal filaments, thus providing a simple explanation why over a time‐course experiment, newly developing oomycete thalli tend to be increasingly lipid‐depleted. Whether this cooperation between algal cells would be mediated by signalling molecules, or simply by the pathogen inducing a source‐sink nutrient gradient across the algal filament, remains open.

### Inducible autophagic processes support oomycete development throughout infection

Over the course of infection, the trophic status of *A. ectocarpii* thalli gradually shifts from lipid‐replete toward lipid‐depleted. Lipid depletion in *A. ectocarpii* was reverted by treatment with autophagy inhibitors, indicative of a role of autophagy in the mobilization of lipid reserves. Similar links between nutrient depletion, the induction of autophagy and sporogenesis are already well‐described in the oomycete *Phytophthora infestans:* using Monodansylcadaverin (MDC) staining, Luo *et al.* (2014) established that autophagosome formation was integral to the development of *P. infestans*, and mostly restricted to zoosporogenesis and cyst germination. In *Phytophthora sojae*, ATG‐related genes were strongly expressed during sporogenesis and infection stages, suggesting a role of autophagy in virulence (Chen *et al.*, 2017).

Lipid depletion correlates with the targeted degradation of some spore initials (sporoptosis), hinting that a secondary response of *Anisolpidium* to nutrient deficiency is the selective autophagy of some spore initials to support the continued development of the others and hence ensure its life cycle completion. This interpretation is consistent with earlier observations that in highly infected brown algae (*Ectocarpus* and *Macrocystis* spp.), some *A. ectocarpii* sporangia contain an odd number of nuclei, despite nuclear divisions always being synchronous throughout the syncytial development of the thallus (Gachon *et al.*, 2017).

Lipid depletion and sporoptosis often links with the development of evaginations of the plasma membrane (EPMs) in the periplasmic space of shrunken thalli (Fig. 6b). Although the role of these enigmatic structures is still unclear, similar structures often called paramural bodies have been implicated in cell wall synthesis in plants (Marchant & Robards, 1968) and fungi (Marchant & Moore, 1973). They proliferate in plants when cell wall appositions are quickly deposited underneath a parasite penetration site (An *et al.*, 2006). The differentiation of EPMs has even been associated to the induction of autophagy in *Arabidopsis* following carbon starvation, strongly suggestive of their involvement in nutrient acquisition (Rose *et al.*, 2006). We were unable to link quantitatively EPM differentiation with lipid depletion in *A. ectocarpii*; however, the presence of conspicuous EPMs in lipid‐depleted thalli suggests that they might have a possible trophic function during *Anisolpidium* starvation.

Although not directly investigated here, it is tempting to speculate that the nutrients remobilized by infected algal cells may be exploited by the oomycete towards its own nutrition (Fig. 6b). Indeed, interference of a pathogen with the autophagy machinery of its host has been reported for example in *Anaplasma phagocytophilum*, an obligatory intracellular bacterium that causes human granulocytic anaplasmosis; infection stimulates host autophagy and re‐direct nutrients to the parasite (Niu *et al.*, 2012). A similar role has been suggested for AWR5, a type III effector of *Ralstonia solanacearum*, which stimulates host autophagy in *Arabidopsis* through target of rapamycin inhibition (Popa *et al.*, 2016). Among oomycetes, *Phytophthora infestans* also releases protein effectors that inhibit the activity of the potato autophagy‐related genes (Dagdas *et al.*, 2016; Maqbool *et al.*, 2016). In the Prymnesiophyte alga *Emiliania huxleyi*, the application of an autophagy inhibitor dramatically reduces infection by the virus EhV201 (Schatz *et al.*, 2014). EhV201 not only takes advantage of the nutrients released through autophagy, but also of the cell membranes, which are needed for building the virion's envelope (Schatz *et al.*, 2014).

### Subversion of pathogen autophagy as a possible algal defence mechanism

Following lipid‐depletion and the induction of targeted sporoptosis, we observed an almost quantitative shift of the *A. ectocarpii* population towards the abortion of entire thalli, a process that prevents the completion of the pathogen's development cycle and thus nullifies its fitness (Fig. 2). Nucleophagy is a hallmark of these entirely abortive pathogen thalli, and is affected by autophagy inhibitors. In yeast, whole nuclei are similarly targeted to vacuoles during sporoptosis, in response to nitrogen and carbon deprivation (Eastwood *et al.*, 2012). Selective nucleophagy also has been described in *Aspergillus oryzae*, which contributes to the recycling of organelles in aging hyphae and supports growth during nutrient deprivation (Shoji *et al.*, 2010).

In *A. ectocarpii*, however, nucleophagy also occurs in abortive syncytia that lack markers of nutrient deprivation (i.e. with reduced EPMs and extensive lipid reserves), suggesting that its induction is disconnected from the nutritional status of *A. ectocarpii*. Therefore, we interpret this abortive nucleophagy as a process of no adaptive value to the parasite, but which benefits the alga. This response is reminiscent of resistant *Nicotiana tabacum*, which triggered ‘vacuolar cell death’ in *Phytophtora parasitica* zoospores, characterized by an external stimulus that led to the production of intracellular reactive oxygen species and ended with pathogen self‐destruction (Galiana *et al.*, 2005). This example shows that subverting the autophagy of the pathogen is a known plant defence strategy that leads to resistance against infection. Therefore, we speculate that in addition to local cell wall reinforcement and pathogen digestion, *M. pyrifera* may subvert the autophagy machinery of *A. ectocarpii* to its benefit, thus efficiently stopping its propagation (Fig. 6c). Interestingly, these abortive *A. ectocarpii* thalli were observed in collapsed algal cells, suggesting that this response constitutes an ultimate line of defence for algal cells otherwise already overcome by infection.

### A possible contribution of inducible host and pathogen autophagic processes to systemic, acquired resistance

The progressive and quantitative shifts observed from nonautophagic to lipid‐depleted and then to wholly abortive thalli strongly suggest that, over time, the host alga acquires increased ability to counteract the pathogen's development. Although we did not attempt to quantify this phenomenon herein, the systemic induction of autophagy in unchallenged cells also may facilitate the early elimination of a newly penetrating *A. ectocarpii* via xenophagy (Fig. 6d), and accordingly lead to another form of systemically increased resistance. We are therefore piloting noninvasive bioassays to measure the evolution of resistance over time at the organismal (as opposed to cellular) level. To the best of our knowledge, acquired resistance to disease has never been reported in macroalgae before, and would have significant agronomical implications. Bearing in mind that clonal algal strains exhibit different levels of innate resistance to infection by *A. ectocarpii* and *E. dicksonii* (Gachon *et al.*, 2009, 2017), this inducible response will be investigated across algal genotypes, which should provide invaluable fundamental knowledge as well as inform future seaweed breeding programmes.

## Author contributions

Original concept (PM, CMMG); algal culturing and experimentation (PM, DGM), microscopy (PM, ME, PvW); statistical analyses (PM); writing the manuscript (PM); editing the manuscript (PM, DGM, ME, PvW, CMMG).

## Supporting information

Please note: Wiley Blackwell are not responsible for the content or functionality of any Supporting Information supplied by the authors. Any queries (other than missing material) should be directed to the *New Phytologist* Central Office.

**Fig. S1** Development cycle of *A. ectocarpii* in its host *M. pyrifera*.
**Fig. S2** Experimental set‐up for the inoculation of *M. pyrifera* with *A. ectocarpii*.
**Fig. S3** Developmental plasticity of *A. ectocarpii* syncytia: autophagy regulates sporogenesis in starved thalli.
**Fig. S4** Lipids accumulate after the exposure to several autophagy inhibitors.
**Fig. S5** MDC signal is induced in *A. ectocarpii* during infection, and disrupted after autophagy inhibitor treatments.
**Fig. S6** Ultrastructural changes undergone by abortive *A. ectocarpii* thalli following autophagy inhibitor treatments (HPF).
**Fig. S7** Representative images illustrating the loss of Lysotracker red signal in *A. ectocarpii* following a 10‐d treatment with autophagy inhibitors.
**Fig. S8** Progressive loss of MDC signal during a 10‐d incubation of *A. ectocarpii* thalli in autophagy inhibitors, using the set‐up described in Fig. S2(c).
**Fig. S9** Host cell wall reinforcement and other cell rearrangements during the *A. ectocarpii* infection course revealed by CHF.
**Fig. S10** Ultrastructure of a mock‐challenged *M. pyrifera* under different TEM techniques.
**Fig. S11** Pattern of MDC staining in mock‐challenged *M. pyrifera* and the sensitivity of inducible MDC staining in unchallenged *M. pyrifera* cells to the application of autophagy inhibitors.
**Fig. S12** Sensitivity of Lysotracker staining in unchallenged host cells to the application of autophagy inhibitors.
**Fig. S13** Ultrastructural changes (CHF) in an unchallenged host cell from a challenged inoculum.
**Fig. S14** TEM evidence for plastid division and accumulation under autophagy inhibitor treatments.
**Fig. S15** Vacuolar–plastid interactions resemble potential chlorophagy‐like autophagy in challenged *M. pyrifera* inoculum.

## References

[nph16438-bib-0001] Alipanah L , Rohloff J , Winge P , Bones AM , Brembu T . 2015 Whole‐cell response to nitrogen deprivation in the diatom *Phaeodactylum tricornutum* . Journal of Experimental Botany 66: 6281–6296. 26163699 10.1093/jxb/erv340 PMC4588885

[nph16438-bib-0002] An Q , Hückelhoven R , Kogel KH , van Bel AJE . 2006 Multivesicular bodies participate in a cell wall‐associated defence response in barley leaves attacked by the pathogenic powdery mildew fungus. Cellular Microbiology 8: 1009–1019. 16681841 10.1111/j.1462-5822.2006.00683.x

[nph16438-bib-0003] Badis Y , Klochkova TA , Strittmatter M , Garvetto A , Murúa P , Sanderson JC , Kim GH , Gachon CMMM . 2019 Novel species of the oomycete *Olpidiopsis* potentially threaten European red algal cultivation. Journal of Applied Phycology 31: 1239–1250.

[nph16438-bib-0004] Bassham DC , Crespo JL . 2014 Autophagy in plants and algae. Frontiers in Plant Science 5: 156–164. 25520731 10.3389/fpls.2014.00679 PMC4248838

[nph16438-bib-0005] Bates D , Mächler M , Bolker B , Walker S . 2015 Fitting linear mixed‐effects models using lme4. Journal of Statistical Software 67: 1–48.

[nph16438-bib-0006] Bouarab K , Potin P , Correa JA , Kloareg B . 1999 Sulfated oligosaccharides mediate the interaction between a marine red alga and its green algal pathogenic endophyte. Plant Cell 11: 1635–1650. 10488232 10.1105/tpc.11.9.1635 PMC144305

[nph16438-bib-0007] Chen L , Zhang X , Wang W , Geng X , Shi Y , Na R , Dou D , Li H . 2017 Network and role analysis of autophagy in *Phytophthora sojae* . Scientific Reports 7: 1879. 28500315 10.1038/s41598-017-01988-7 PMC5431975

[nph16438-bib-0008] Cock JM , Sterck L , Rouzé P , Scornet D , Allen AE , Amoutzias G , Anthouard V , Artiguenave F , Aury J‐M , Badger JH *et al* 2010 The *Ectocarpus* genome and the independent evolution of multicellularity in brown algae. Nature 465: 617–621. 20520714 10.1038/nature09016

[nph16438-bib-0009] Coelho SM , Scornet D , Rousvoal S , Peters N , Dartevelle L , Peters AF , Cock JM . 2012 Isolation and regeneration of protoplasts from *Ectocarpus* . Cold Spring Harbor Protocols 7: 361–364. 10.1101/pdb.prot067959 22383637

[nph16438-bib-0010] Dagdas YF , Beihaj K , Maqbool A , Chaparro‐Garcia A , Pandey P , Petre B , Tabassum N , Cruz‐Mireles N , Hughes RK , Sklenar J *et al* 2016 An effector of the Irish potato famine pathogen antagonizes a host autophagy cargo receptor. eLife 5: 1–23. 10.7554/eLife.10856 PMC4775223 26765567

[nph16438-bib-0011] Dagdas YF , Pandey P , Tumtas Y , Sanguankiattichai N , Belhaj K , Duggan C , Leary AY , Segretin M , Contreras M , Savage Z *et al* 2018 Host autophagy machinery is diverted to the pathogen interface to mediate focal defense responses against the Irish potato famine pathogen. eLife 7: e37476. 29932422 10.7554/eLife.37476 PMC6029844

[nph16438-bib-0013] Dittami SM , Scornet D , Petit J‐L , Ségurens B , Da Silva C , Corre E , Dondrup M , Glatting K‐H , König R , Sterck L *et al* 2009 Global expression analysis of the brown alga *Ectocarpus siliculosus* (Phaeophyceae) reveals large‐scale reprogramming of the transcriptome in response to abiotic stress. Genome Biology 10: R66. 19531237 10.1186/gb-2009-10-6-r66 PMC2718500

[nph16438-bib-0014] Eastwood MD , Cheung SWT , Lee KY , Moffat J , Meneghini MD . 2012 Developmentally programmed nuclear destruction during yeast gametogenesis. Developmental Cell 23: 35–44. 22727375 10.1016/j.devcel.2012.05.005

[nph16438-bib-0012] de Figueiredo P , Dickman M . 2016 Autophagy under attack. eLife 5: e14447. 26906167 10.7554/eLife.14447 PMC4775212

[nph16438-bib-0015] Gachon CMM , Strittmatter M , Badis Y , Fletcher KI , Van West P , Müller DG . 2017 Pathogens of brown algae: culture studies of *Anisolpidium ectocarpii* and *A. rosenvingei* reveal that the Anisolpidiales are uniflagellated oomycetes. European Journal of Phycology 52: 133–148.

[nph16438-bib-0016] Gachon CMM , Strittmatter M , Muller DG , Kleinteich J , Kupper FC , Müller DG , Kleinteich J , Küpper FC . 2009 Detection of differential host susceptibility to the marine oomycete pathogen *Eurychasma dicksonii* by real‐time PCR: not all algae are equal. Applied and Environmental Microbiology 75: 322–328. 19011072 10.1128/AEM.01885-08 PMC2620704

[nph16438-bib-0017] Galiana E , Rivière MP , Pagnotta S , Baudouin E , Panabières F , Gounon P , Boudier L . 2005 Plant‐induced cell death in the oomycete pathogen *Phytophthora parasitica* . Cellular Microbiology 7: 1365–1378. 16098223 10.1111/j.1462-5822.2005.00565.x

[nph16438-bib-0018] Garvetto A , Nézan E , Badis Y , Bilien G , Arce P , Bresnan E , Gachon CMM , Siano R . 2018 Novel widespread marine oomycetes parasitising diatoms, including the toxic genus *Pseudo‐nitzschia*: genetic, morphological, and ecological characterisation. Frontiers in Microbiology 9: 2918. 30559730 10.3389/fmicb.2018.02918 PMC6286980

[nph16438-bib-0019] Hurd CL , Harrison PJ , Bischof K , Lobban CS . 2014 Seaweed ecology and physiology. Cambridge, UK: Cambridge University Press.

[nph16438-bib-0020] Klionsky DJ , Abdelmohsen K , Abe A , Abedin MJ , Abeliovich H , Acevedo Arozena A , Adachi H , Adams CM , Adams PD , Adeli K *et al* 2016 Guidelines for the use and interpretation of assays for monitoring autophagy. Autophagy 12: 1–222. 26799652 10.1080/15548627.2015.1100356 PMC4835977

[nph16438-bib-0021] Klochkova TA , Shin YJ , Moon KH , Motomura T , Kim GH . 2016 New species of unicellular obligate parasite, *Olpidiopsis pyropiae* sp. nov., that plagues Pyropia sea farms in Korea. Journal of Applied Phycology 28: 73–83.

[nph16438-bib-0022] Krumhansl KA , Okamoto DK , Rassweiler A , Novak M , Bolton JJ , Cavanaugh KC , Connell SD , Johnson CR , Konar B , Ling SD *et al* 2016 Global patterns of kelp forest change over the past half‐century. Proceedings of the National Academy of Sciences, USA 113: 13785–13790. 10.1073/pnas.1606102113 PMC5137772 27849580

[nph16438-bib-0023] Küpper FC , Carpenter LJ , McFiggans GB , Palmer CJ , Waite TJ , Boneberg E‐M , Woitsch S , Weiller M , Abela R , Grolimund D *et al* 2008 Iodide accumulation provides kelp with an inorganic antioxidant impacting atmospheric chemistry. Proceedings of the National Academy of Sciences, USA 105: 6954–6958. 10.1073/pnas.0709959105 PMC2383960 18458346

[nph16438-bib-0024] Küpper FC , Gaquerel E , Cosse A , Adas F , Peters AF , Müller DG , Kloareg B , Salaün J‐P , Potin P . 2009 Free fatty acids and methyl jasmonate trigger defense reactions in *Laminaria digitata* . Plant and Cell Physiology 50: 789–800. 19213737 10.1093/pcp/pcp023

[nph16438-bib-0025] Küpper FC , Kloareg B , Guern J , Potin P . 2001 Oligoguluronates elicit an oxidative burst in the brown algal kelp *Laminaria digitata* . Plant Physiology 125: 278–291. 11154336 10.1104/pp.125.1.278 PMC61009

[nph16438-bib-0026] Lenz HD , Haller E , Melzer E , Kober K , Wurster K , Stahl M , Bassham DC , Vierstra RD , Parker JE , Bautor J *et al* 2011 Autophagy differentially controls plant basal immunity to biotrophic and necrotrophic pathogens. The Plant Journal 66: 818–830. 21332848 10.1111/j.1365-313X.2011.04546.x

[nph16438-bib-0027] Luo Q , Wang FX , Zhong NQ , Wang HY , Xia GX . 2014 The role of autophagy during development of the oomycete pathogen *Phytophthora infestans* . Journal of Genetics and Genomics 41: 225–228. 24780621 10.1016/j.jgg.2014.03.004

[nph16438-bib-0028] Maqbool A , Hughes RK , Dagdas YF , Tregidgo N , Zess E , Belhaj K , Round A , Bozkurt TO , Kamoun S , Banfield MJ . 2016 Structural basis of host autophagy‐related protein 8 (ATG8) binding by the Irish potato famine pathogen effector protein PexRD54. Journal of Biological Chemistry 291: 20270–20282. 27458016 10.1074/jbc.M116.744995 PMC5025708

[nph16438-bib-0029] Marchant R , Moore RT . 1973 Lomasomes and plasmalemmasomes in fungi. Protoplasma 76: 235–247.

[nph16438-bib-0030] Marchant R , Robards AW . 1968 Membrane systems associated with the plasmalemma of plant cells. Annals of Botany 32: 457–471.

[nph16438-bib-0031] Melendez A , Neufeld TP . 2008 The cell biology of autophagy in metazoans: a developing story. Development 135: 2347–2360. 18567846 10.1242/dev.016105 PMC2702844

[nph16438-bib-0032] Minina EA , Bozhkov PV , Hofius D . 2014 Autophagy as initiator or executioner of cell death. Trends in Plant Science 19: 692–697. 25156061 10.1016/j.tplants.2014.07.007

[nph16438-bib-0033] Monier A , Chambouvet A , Milner DS , Attah V , Terrado R , Lovejoy C , Moreau H , Santoro AE , Derelle É , Richards TA . 2017 Host‐derived viral transporter protein for nitrogen uptake in infected marine phytoplankton. Proceedings of the National Academy of Sciences, USA 114: E7489–E7498. 10.1073/pnas.1708097114 PMC5594687 28827361

[nph16438-bib-0034] Murúa P . 2018 Molecular and cell biology of novel brown algal pathosystems. PhD thesis, University of Aberdeen, Aberdeen, UK.

[nph16438-bib-0035] Murúa P , Goecke F , Westermeier R , van West P , Küpper FC , Neuhauser S . 2017 *Maullinia braseltonii* sp. nov. (Rhizaria, Phytomyxea, Phagomyxida): A cyst‐forming parasite of the bull kelp *Durvillaea* spp. (Stramenopila, Phaeophyceae, Fucales). Protist 168: 468–480. 28822911 10.1016/j.protis.2017.07.001 PMC5673062

[nph16438-bib-0036] Niu H , Xiong Q , Yamamoto A , Hayashi‐Nishino M , Rikihisa Y . 2012 Autophagosomes induced by a bacterial Beclin 1 binding protein facilitate obligatory intracellular infection. Proceedings of the National Academy of Sciences, USA 109: 20800–20807. 10.1073/pnas.1218674109 PMC3529060 23197835

[nph16438-bib-0037] Oliveira L , Bisalputra T . 1977 Ultrastructural and cytochemical studies on the nature and origin of the cytoplasmic inclusions of aging cells of *Ectocarpus* (Phaeophyta, Ectocarpales). Phycologia 16: 235–243.

[nph16438-bib-0038] Pavia H , Baumgartner F , Cervin G , Enge S , Kubanek J , Nylund GM , Selander E , Svensson JR , Toth GB . 2012 Chemical defences against herbivores In: Bronmark C, Hansson L‐A, eds. Chemical ecology in aquatic systems. Oxford, UK: Oxford University Press, 210–235.

[nph16438-bib-0039] Pellegrini L . 1979 On the origin and development of vacuoles in promeristematic cells of *Cystoseira stricta* Sauvageau (Phaeophyta, Fucales). Protoplasma 101: 89–102.

[nph16438-bib-0040] Popa C , Li L , Gil S , Tatjer L , Hashii K , Tabuchi M , Coll NS , Ariño J , Valls M . 2016 The effector AWR5 from the plant pathogen *Ralstonia solanacearum* is an inhibitor of the TOR signalling pathway. Scientific Reports 6: 27058. 27257085 10.1038/srep27058 PMC4891724

[nph16438-bib-0041] Ritter A , Dittami SM , Goulitquer S , Correa JA , Boyen C , Potin P , Tonon T . 2014 Transcriptomic and metabolomic analysis of copper stress acclimation in *Ectocarpus siliculosus* highlights signaling and tolerance mechanisms in brown algae. BMC Plant Biology 14: 116. 24885189 10.1186/1471-2229-14-116 PMC4108028

[nph16438-bib-0042] Rose TL , Bonneau L , Der C , Marty‐Mazars D , Marty F . 2006 Starvation‐induced expression of autophagy‐related genes in *Arabidopsis* . Biology of the Cell 98: 53–67. 16354162 10.1042/BC20040516

[nph16438-bib-0043] Schatz D , Shemi A , Rosenwasser S , Sabanay H , Wolf SG , Ben‐Dor S , Vardi A . 2014 Hijacking of an autophagy‐like process is critical for the life cycle of a DNA virus infecting oceanic algal blooms. New Phytologist 204: 854–863. 25195618 10.1111/nph.13008 PMC4233938

[nph16438-bib-0044] Segovia M . 2003 Cell death in the unicellular Chlorophyte *Dunaliella tertiolecta*. A hypothesis on the evolution of apoptosis in higher plants and metazoans. Plant Physiology 132: 99–105. 12746516 10.1104/pp.102.017129 PMC166956

[nph16438-bib-0045] Shemi A , Ben‐Dor S , Vardi A . 2015 Elucidating the composition and conservation of the autophagy pathway in photosynthetic eukaryotes. Autophagy 11: 701–715. 25915714 10.1080/15548627.2015.1034407 PMC4502668

[nph16438-bib-0046] Shoji J , Kikuma T , Arioka M , Kitamoto K . 2010 Macroautophagy‐mediated degradation of whole nuclei in the filamentous fungus *Aspergillus oryzae* . PLoS ONE 5: e15650. 21187926 10.1371/journal.pone.0015650 PMC3004950

[nph16438-bib-0047] Shpilka T , Welter E , Borovsky N , Amar N , Shimron F , Peleg Y , Elazar Z . 2015 Fatty acid synthase is preferentially degraded by autophagy upon nitrogen starvation in yeast. Proceedings of the National Academy of Sciences, USA 112: 1434–1439. 10.1073/pnas.1409476112 PMC4321280 25605918

[nph16438-bib-0048] Strittmatter M , Gachon CMM , Müller DG , Kleinteich J , Heesch S , Tsirigoti A , Katsaros C , Kostopoulou M , Küpper FC . 2013 Intracellular eukaryotic pathogens in brown macroalgae in the Eastern Mediterranean, including LSU rRNA data for the oomycete *Eurychasma dicksonii* . Diseases of Aquatic Organisms 104: 1–11. 23670075 10.3354/dao02583

[nph16438-bib-0049] Thomas F , Cosse A , Goulitquer S , Raimund S , Morin P , Valero M , Leblanc C , Potin P . 2011 Waterborne signaling primes the expression of elicitor‐induced genes and buffers the oxidative responses in the brown alga *Laminaria digitata* . PLoS ONE 6: 1–12. 10.1371/journal.pone.0021475 PMC3123347 21731761

[nph16438-bib-0050] Thomas F , Cosse A , Le Panse S , Kloareg B , Potin P , Leblanc C . 2014 Kelps feature systemic defense responses: Insights into the evolution of innate immunity in multicellular eukaryotes. New Phytologist 204: 567–576. 25041157 10.1111/nph.12925

[nph16438-bib-0051] Tsirigoti A , Beakes GW , Hervé C , Gachon CMM , Katsaros C . 2015 Attachment, penetration and early host defense mechanisms during the infection of filamentous brown algae by *Eurychasma dicksonii* . Protoplasma 252: 845–856. 25385261 10.1007/s00709-014-0721-1

[nph16438-bib-0052] Wickham H . 2009 ggplot2. New York, NY, USA: Springer New York.

[nph16438-bib-0053] Zambounis A , Elias M , Sterck L , Maumus F , Gachon CMM . 2012 Highly dynamic exon shuffling in candidate pathogen receptors … What if brown algae were capable of adaptive immunity? Molecular Biology and Evolution 29: 1263–1276. 22144640 10.1093/molbev/msr296 PMC3341825

