# Supplementary material for: Host and pathogen autophagy are central to the inducible local defences and systemic response of the giant kelp Macrocystis pyrifera against the oomycete pathogen Anisolpidium ectocarpii
Source: New Phytol. 2020 Feb 29;226(5):1445–60. doi: 10.1111/nph.16438 (PMC7317505; doi:10.1111/nph.16438)

New Phytologist

***New Phytologist* Supporting Information**

Article title: **Host and pathogen autophagy are central to the inducible local defences and systemic response of the giant kelp *Macrocystis pyrifera* against the oomycete pathogen *Anisolpidium ectocarpii***

Authors: Pedro Murúa, Dieter G. Müller, Mohammad Etemadi, Pieter van West, Claire M. M. Gachon

Article acceptance date: 08 January 2020

The following Supporting Information is available for this article:

Fig. S1 **Development cycle of *Anisolpidium ectocarpii* in its host *Macrocystis pyrifera.***

Fig. S2 **Experimental set-up for the inoculation of *Macrocystis pyrifera* with *A. ectocarpii*.**

Fig. S3 **Developmental plasticity of *A. ectocarpii* syncytia: autophagy regulates sporogenesis in starved thalli.**

Fig. S4 **Lipids accumulate after the exposure to several autophagy inhibitors.**

Fig. S5 **MDC signal is induced in *A. ectocarpii* during infection, and disrupted after autophagy inhibitor treatments.**

Fig. S6 **Ultrastructural changes undergone by abortive *A. ectocarpii* thalli following autophagy inhibitor treatments (HPF).**

Fig. S7 **Representative images illustrating the loss of Lysotracker red signal in *A. ectocarpii* following a 10-day treatment with autophagy inhibitors.**

Fig. S8 **Progressive loss of MDC signal during a 10-day incubation of *A. ectocarpii* thalli in autophagy inhibitors, using the set-up described in Suppl. Fig. 2c.**

Fig. S9 **Host cell wall reinforcement and other cell rearrangements during the *A. ectocarpii* infection course revealed by CHF.**

Fig. S10 **Ultrastructure of a mock-challenged *Macrocystis pyrifera* under different TEM techniques.**

Fig. S11 **Pattern of MDC staining in mock-challenged *M. pyrifera* and the sensitivity of inducible MDC staining in unchallenged *M. pyrifera* cells to the application of autophagy inhibitors.**

Fig. S12 **Sensitivity of Lysotracker staining in unchallenged host cells to the application of autophagy inhibitors.**

Fig. S13 **Ultrastructural changes (CHF) in an unchallenged host cell from a challenged inoculum.**

Fig. S14 **TEM evidence for plastid division and accumulation under autophagy inhibitor treatments.**

Fig. S15 **Vacuolar-plastid interactions resemble potential chlorophagy-like autophagy in challenged *M. pyrifera* inoculum.**

Fig. S1 Development cycle of *Anisolpidium ectocarpii* in its host *Macrocystis pyrifera.* (a) Infection by *A. ectocarpii* starts by the encystment of spores on *M. pyrifera* filament (arrows). Scale bar: 10 µm. (b) Development of unwalled *A. ectocarpii* syncytia (arrowheads) inside vegetative cells of *M. pyrifera*. Scale bar: 12 µm. (c) A cell wall is developed when growth stops, giving a typical circular to ellipsoidal shape to the *A. ectocarpii* syncytium (arrowheads). Sometimes, syncytia are vacuolated (double arrowhead). NB: for clarity, not all visible pathogen thalli are pointed. Scale bar: 16 µm. (d) After maturation, *A. ectocarpii* develops an exit tube (arrow, also called discharge tube), through which the zoospores are released, leaving an empty sporangium behind. Scale bar: 15 µm. (e) Unwalled *A. ectocarpii* under CHF TEM. N: nucleus; v: vacuole; m: mitochondrion; PM: plasma membrane; HCW: host cell wall; HCy: host cytosol. Scale bar: 500 nm. (f) Walled *A. ectocarpii* under CHF TEM. N: nucleus; CW cell wall; Cd: host debris in the cytosol; HCW: host cell wal. Scale bar: 1 µm.

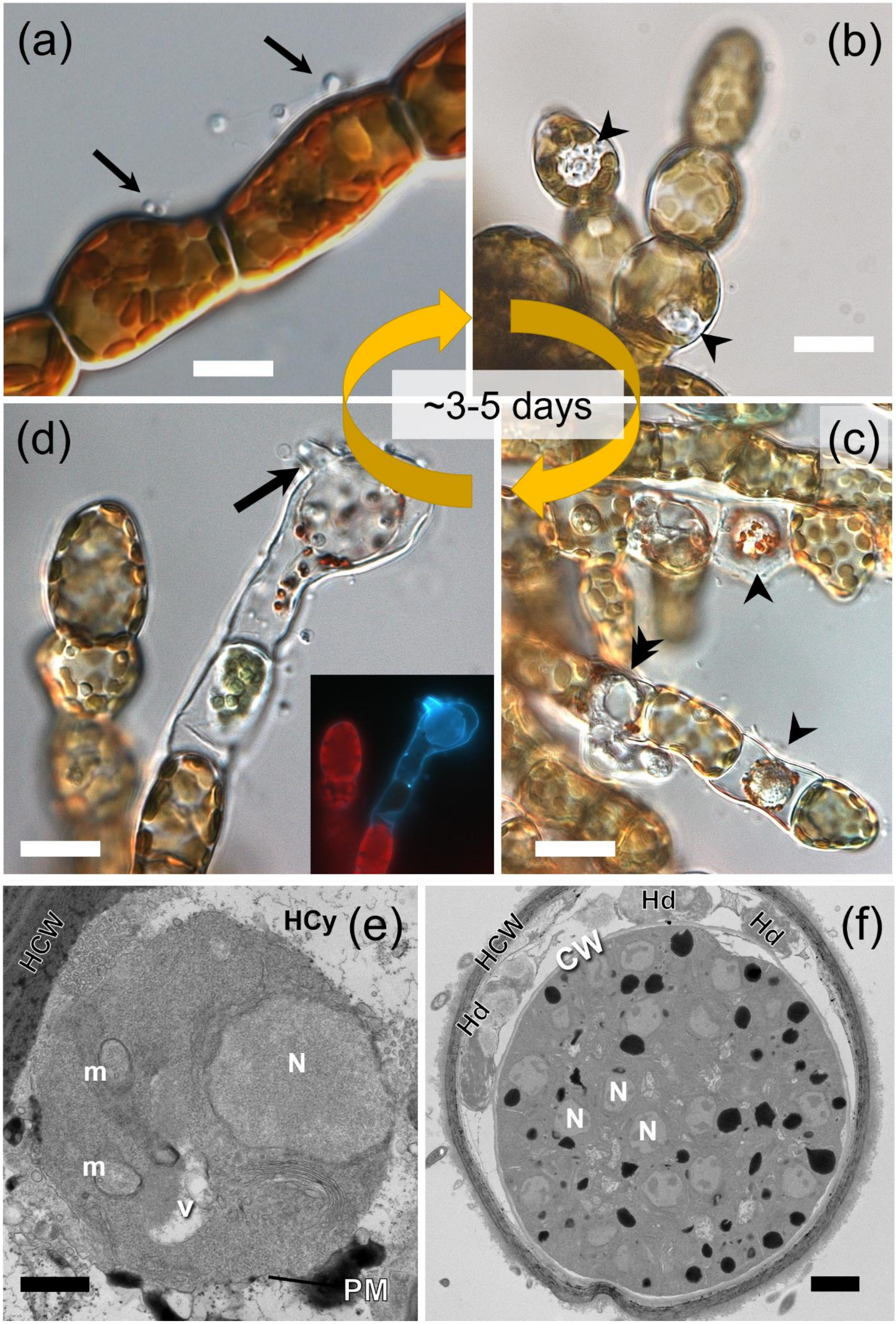

(a)
(b)
~3-5 days
(d)
(c)
HCW
HCy
(e)
m
N
m
v
PM
Hd
Hd
HCW
CW
(f)
Hd
N
N
N

Fig. S2 Experimental set-up for the inoculation of *Macrocystis pyrifera* with *A. ectocarpii*.

(a) *A. ectocarpii* is an obligate biotroph pathogen that cannot be maintained outside its host. To perform inoculations, *Anisolpidium*-infected *M. pyrifera* tufts were placed in 70-µm cell strainers that allow the free passage of pathogen spores into the surrounding medium containing the target alga *M. pyrifera*. To perform "mock-challenged controls", healthy *M. pyrifera* was placed in the strainers instead of infected tufts, and the target alga was examined. (b) Within the target - originally healthy- algal tuft, the population of host cells is further classified as i) infected (containing one pathogen thallus or more), ii) challenged (at least one encysted *A. ectocarpii* spores on the surface (blue arrowheads) with or without a detectable thallus inside the cell) or iii) unchallenged cells (with no evidence of direct interaction with *A. ectocarpii* spores, and no evidence of an intracellular thallus). (c) Target *Macrocystis* was sampled within 17 dai for time series experiment. In order to check the effect of autophagy inhibitors, this 17 dai material was transferred to new PES medium with the respective inhibitor, where it was incubated for 10 additional days.

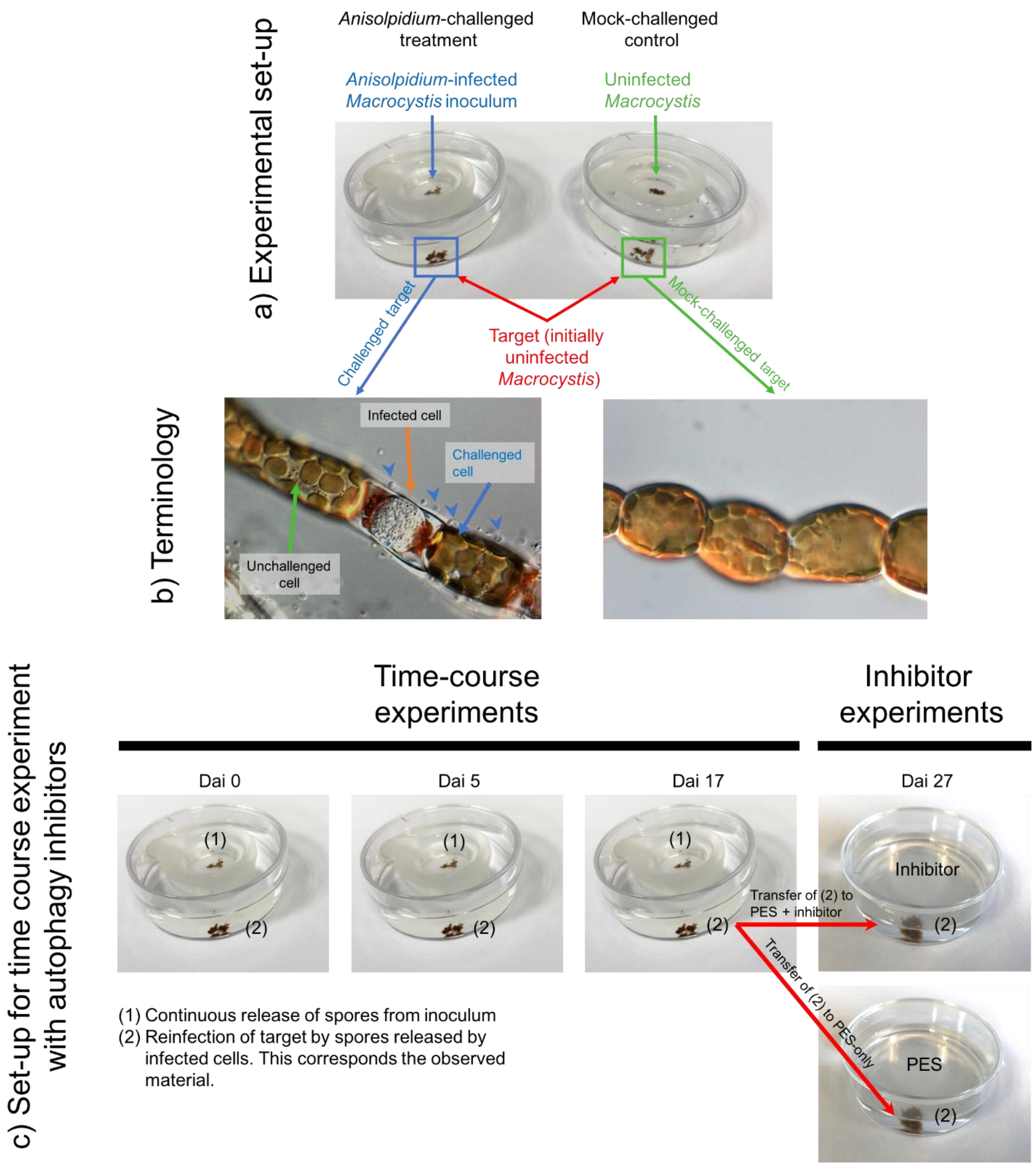
a) Experimental set-up
Anisolpidium-challenged treatment
Mock-challenged control
Anisolpidium-infected *Macrocystis* inoculum
Uninfected *Macrocystis*
Challenged target
Mock-challenged target
Target (initially uninfected *Macrocystis*)
b) Terminology
Infected cell
Challenged cell
Unchallenged cell
c) Set-up for time course experiment with autophagy inhibitors
Time-course experiments
Inhibitor experiments
Dai 0
Dai 5
Dai 17
Dai 27
(1)
(2)
Inhibitor
PES
Transfer of (2) to PES + inhibitor
Transfer of (2) to PES-only
(1) Continuous release of spores from inoculum
(2) Reinfection of target by spores released by infected cells. This corresponds the observed material.

Fig. S3 Developmental plasticity of *A. ectocarpii* syncytia: autophagy regulates sporogenesis in starved thalli. NB: pathogen and host structures are annotated in white and black, respectively. (a) *M. pyrifera* infected by *A. ectocarpii* under DIC microscopy, showing vacuolated (double arrowhead) and non-vacuolated thalli (arrowhead). Scale bar: 12 μm. (b) *A. ectocarpii* syncytium before cleavage (Sy), under HPF TEM. CW: Pathogen's cell wall. HCW: Host cell wall; HCy: Host cytosol; HPM: Host plasma membrane. Scale bar: 2 μm. (c) Magnification of an unsegmented syncytium of *A. ectocarpii* from image (b) (inset (c)). N: Nucleus; Lg: Lipid globule; V: vacuole; M: mitochondrion; Arrowheads: microbody-like structures. Scale bar: 500 nm. (d) Additional (rare) evidence of piecemeal digestion (arrowhead) by burgeoning of the nucleus (N), under HPF TEM. Scale bar: 500 nm. (e) Magnified periplasmic space area from image (b) (inset (e)), showing the early development of tubular membranar structures underneath the cell wall of *A. ectocarpii*. Scale bar: 2 μm. (f) A lipid-rich *A. ectocarpii* syncytium (Sy) in a singly-infected host cell fills completely the space within its delimiting cell wall and accumulates numerous dark lipid globules (CHF). CW: *A. ectocarpii* cell wall; Hd: host cell debris; HCW: host cell wall; HCy: host cytosol; HPM: residual host plasma membrane. Scale bar: 2 μm. (g) Magnification of a margin of the syncytium shown in (f) (inset (g)), pointing out a small evagination of the plasma membrane (EPM) close to the pathogen cell wall (CW). Hd: Host cell debris. Scale bar: 500 nm. (h) Magnification of a segmenting syncytium of *A. ectocarpii* from image (f) (inset (h)), pointing the cleaving areas (arrowheads). N: Nucleus; Lg: Lipid globule; M: mitochondrion; GA: Golgi apparatus. Scale bar: 500 nm. (i) CHF microphotograph of a shrunken *A. ectocarpii* syncytia (Sy) in a multiply-infected host cell, with characteristic prominent periplasmic spaces (double arrowheads). HCW: Host cell wall; eSy: Empty *A. ectocarpii* sporangium. Scale bar: 2 μm. (j) Magnification of a shrunken syncytium of *A. ectocarpii* (Sy) from image i (inset (j)). EPM: evagination of the plasma membrane.

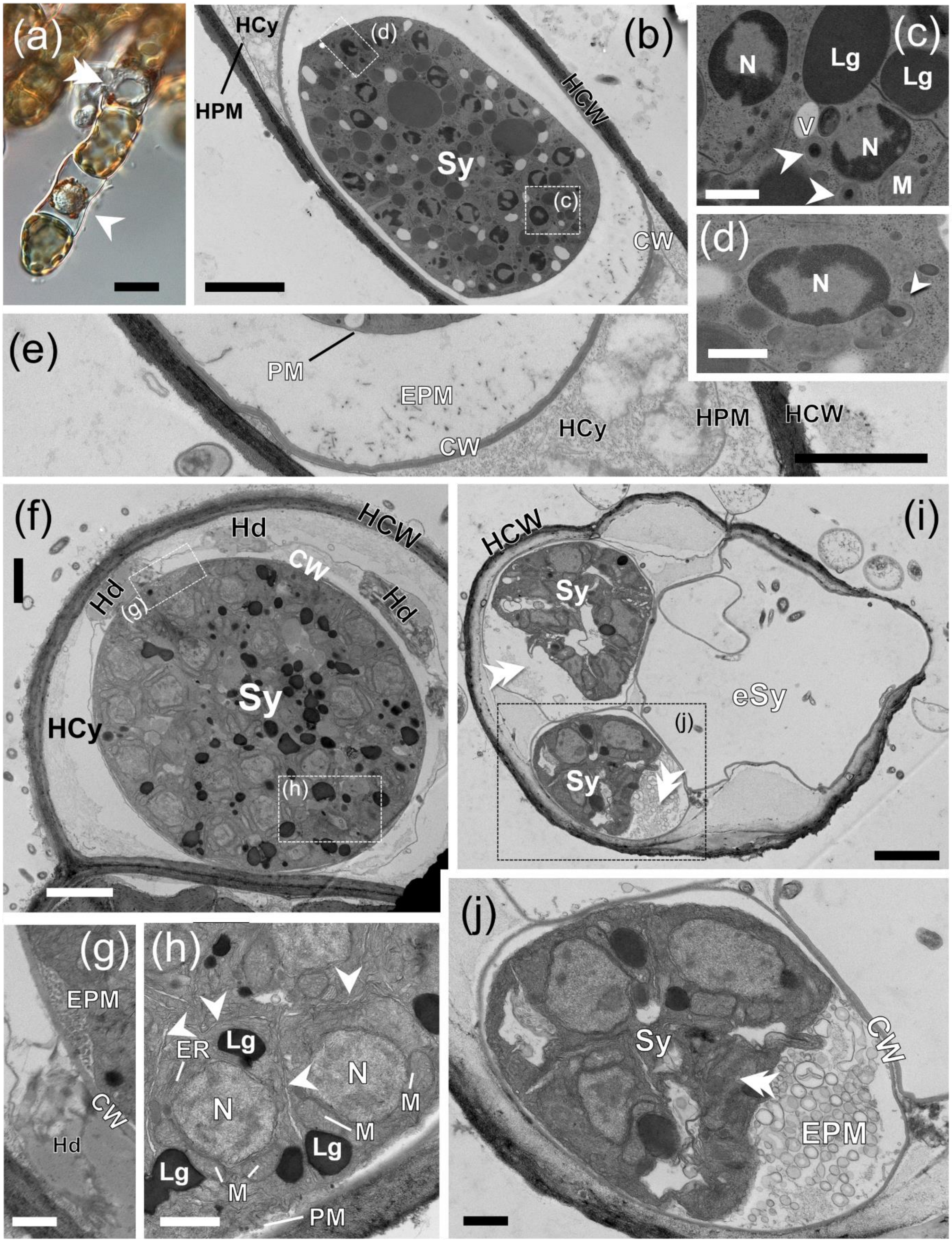

(a)
(b)
HCy
HPM
HCW
Sy
CW
(d)
(c)
N
Lg
Lg
V
N
M
(d)
N
(e)
PM
EPM
HCy
HPM
HCW
CW
(f)
Hd
HCW
CW
Hd
Hd
(g)
Sy
HCy
(h)
(i)
HCW
Sy
eSy
(j)
Sy
(g)
EPM
CW
Hd
(h)
ER
Lg
N
N
M
M
Lg
Lg
M
PM
(j)
Sy
CW
EPM

Fig. S4 Lipids accumulate after the exposure to several autophagy inhibitors.

Representative lipid-related phenotypes observed under different autophagy inhibitors. Scale bars: 16 µm. Arrowheads: *A. ectocarpii* thalli. Note that BODIPY also stains the surface bacteria.

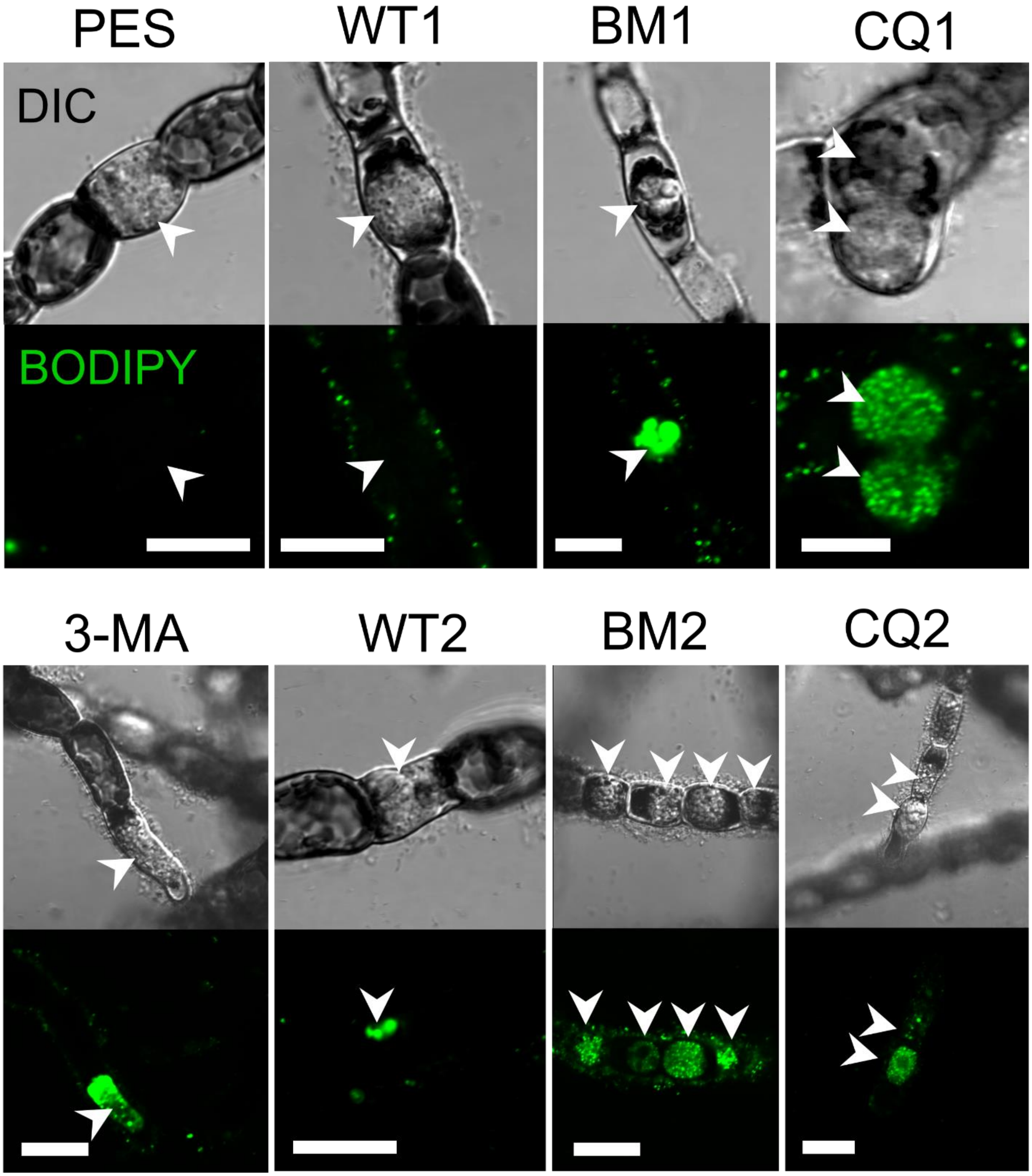

Fig. S5 MDC signal is induced in *A. ectocarpii* during infection, and disrupted after autophagy inhibitor treatments. (a) MDC signal increase in a time-course experiment of 20 dai (days after infection), in comparison with a mock-infected control. Scale bar: 100 μm. (b) Comparison of autophagic (arrowhead) and non-autophagic (double arrowhead) *A. ectocarpii* syncytia by MDC, 5 dai. Scale bar: 15 μm. (c) Autophagic *A. ectocarpii* syncytia detected by MDC in a densely-infected *M. pyrifera* filament, 15 dai. Note that MDC does not allow to discriminate between “starving” and “abortive” autophagic thalli. Scale bar: 15 μm. (d) Accumulation of autophagic thalli in the *A. ectocarpii* population, as measured with MDC. Individual time courses are shown for 8 replicates. Time points where MDC-positive *A. ectocarpii* were not found are designated as magenta dots. Letters on every sampling day designate the statistically significant differences between time-points (multiple comparisons, Friedman tests), where a < b and p<0.05. (e) Ratio of MDC-positive *A. ectocarpii* thalli over the total number of pathogen thalli, following a 10-day exposure to different autophagy inhibitors. PES: Control without inhibitor (Provasoli-enriched seawater medium); WT1 and WT2: 250 nM and 1 μM Wortmannin; 3-MA: 10 mM 3-Methyladenine; BM1 and BM2: 0.1 and 0.5 μM Bafilomycin. CQ1 and CQ2: 50 and 200 μM Chloroquine. Letters on every sampling day designate the statistically significant differences between time-points (LMM), where a < b < c (p<0.05), after Tukey test for multiple comparisons. (f) Autophagy inhibitors contributed to abolish/reduce the extent of MDC positive *Anisolpidium* thalli in *M. pyrifera* after 10 days of exposure. Scale bar: 15 μm.

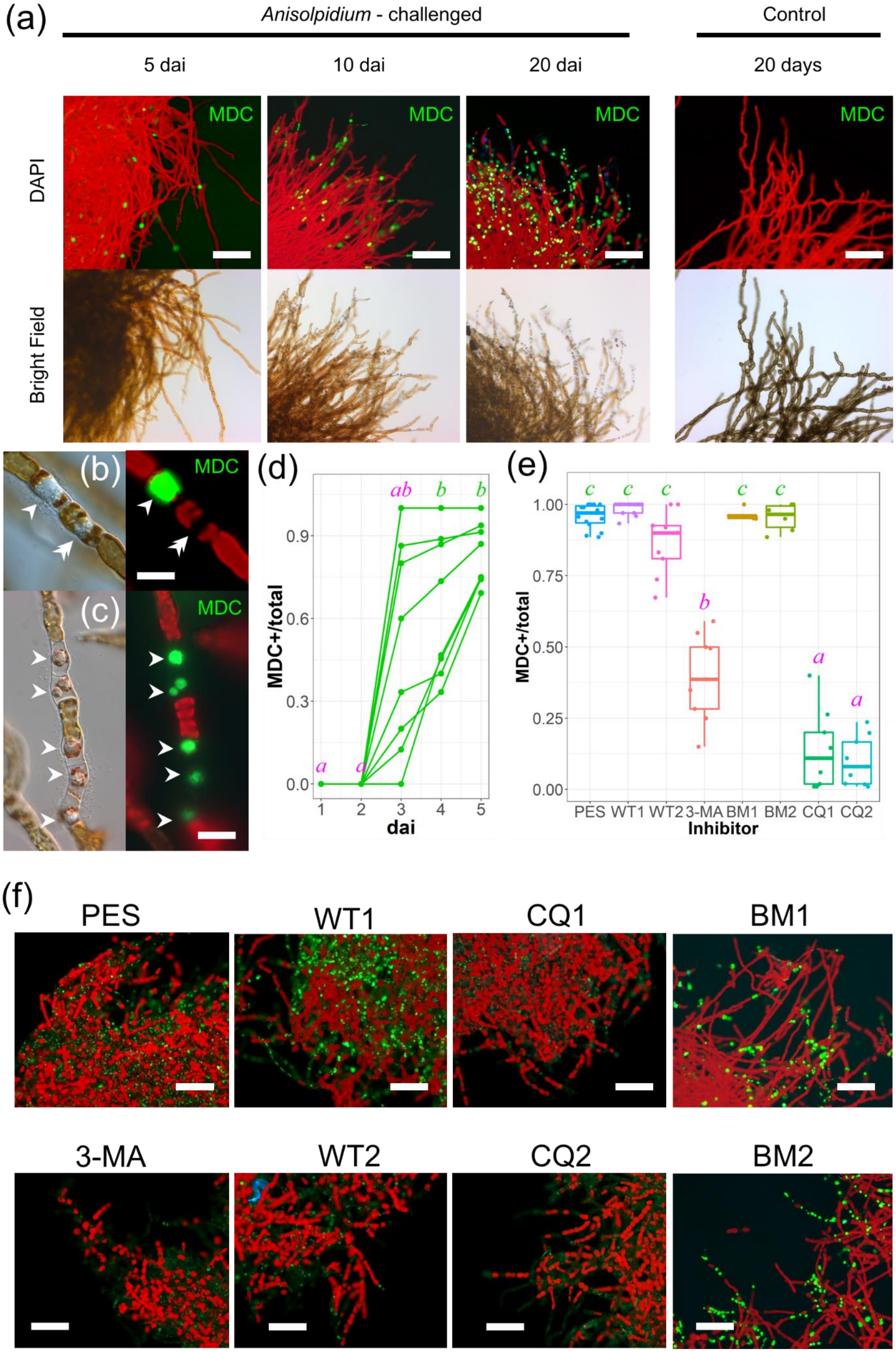

(a)
Anisolpidium - challenged
Control
5 dai
10 dai
20 dai
20 days
DAPI
Bright Field
MDC
(b)
(c)
(d)
MDC+/total
dai
(e)
MDC+/total
Inhibitor
PES
WT1
WT2
3-MA
BM1
BM2
CQ1
CQ2
(f)

Fig. S6 Ultrastructural changes undergone by abortive *A. ectocarpii* thalli following autophagy inhibitor treatments (HPF) (a) Magnification of the abortive syncytium (inset on Fig. 2d), showing the presence of vacuoles filled with unrecognisable cell debris (arrowhead), despite the relative abundance of lipid globules (Lg). Double arrowhead: vacuoles containing condensed nuclei (refer to Suppl. Fig. 3c for a comparison of nuclei under HPF). CW: cell wall. (b) Magnification of the late autophagic syncytium showed in inset S6b on Fig. 2e, highlighting the presence of vacuoles engulfing cellular structures and digestive vesicles filled with cellular debris. (c) Peripheral vesicles (double arrowhead) are also extensively seen in the late autophagic stage shown in Fig. 2e (inset S6c). (d) – (g) Treatments with wortmannin WT1 (d – e) and WT2 (f) – (g) normally showed highly vacuolated *A. ectocarpii* syncytia, with nuclei (N) inside vacuoles. Additionally, syncytia tend to accumulate lipid droplets in the cytosol (arrows). (h) – (i) Following incubation with 3-MA, *A. ectocarpii* syncytia were very vacuolated but no recognisable digestion of any organelles was recorded. (j) – (m) Treatments with bafilomycin BM1 (j – k) and BM2 (l – n) showed intact nuclei (N) and also an important accumulation of lipids in *A. ectocarpii* (arrows; not all of them are labelled to avoid overloading the figure); small double membrane vesicles resembling autophagosomes were relatively abundant (ap). (n) – (q) Incubations with chloroquine (CQ1: image n) led to the accumulation of lipids, intact nuclei (N); at higher concentration (CQ2: images p – q), thalli contained vacuoles that accumulated debris. (q) Although less frequent than in the bafilomycin treatments, some double-membrane structures (ap) were present in the cytosol. Scale bars: 1 μm, except for c: 500 nm; k, m, q: 100 nm.

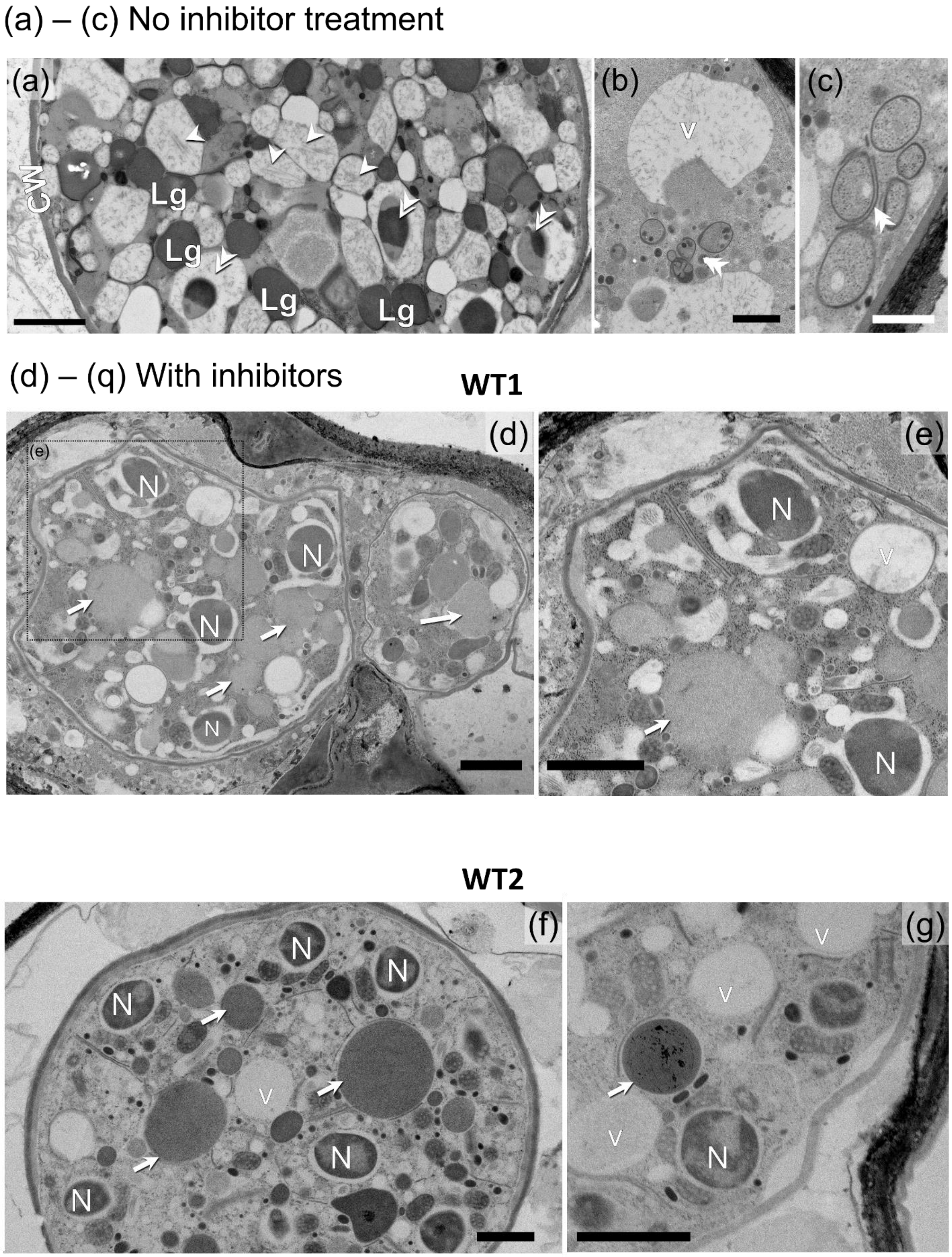
(a) – (c) No inhibitor treatment
(a)
CW
Lg
Lg
Lg
Lg
(b)
V
(c)
(d) – (q) With inhibitors
WT1
(d)
(e)
N
N
N
N
(e)
N
V
N
WT2
(f)
N
N
N
V
N
N
(g)
V
V
V
N

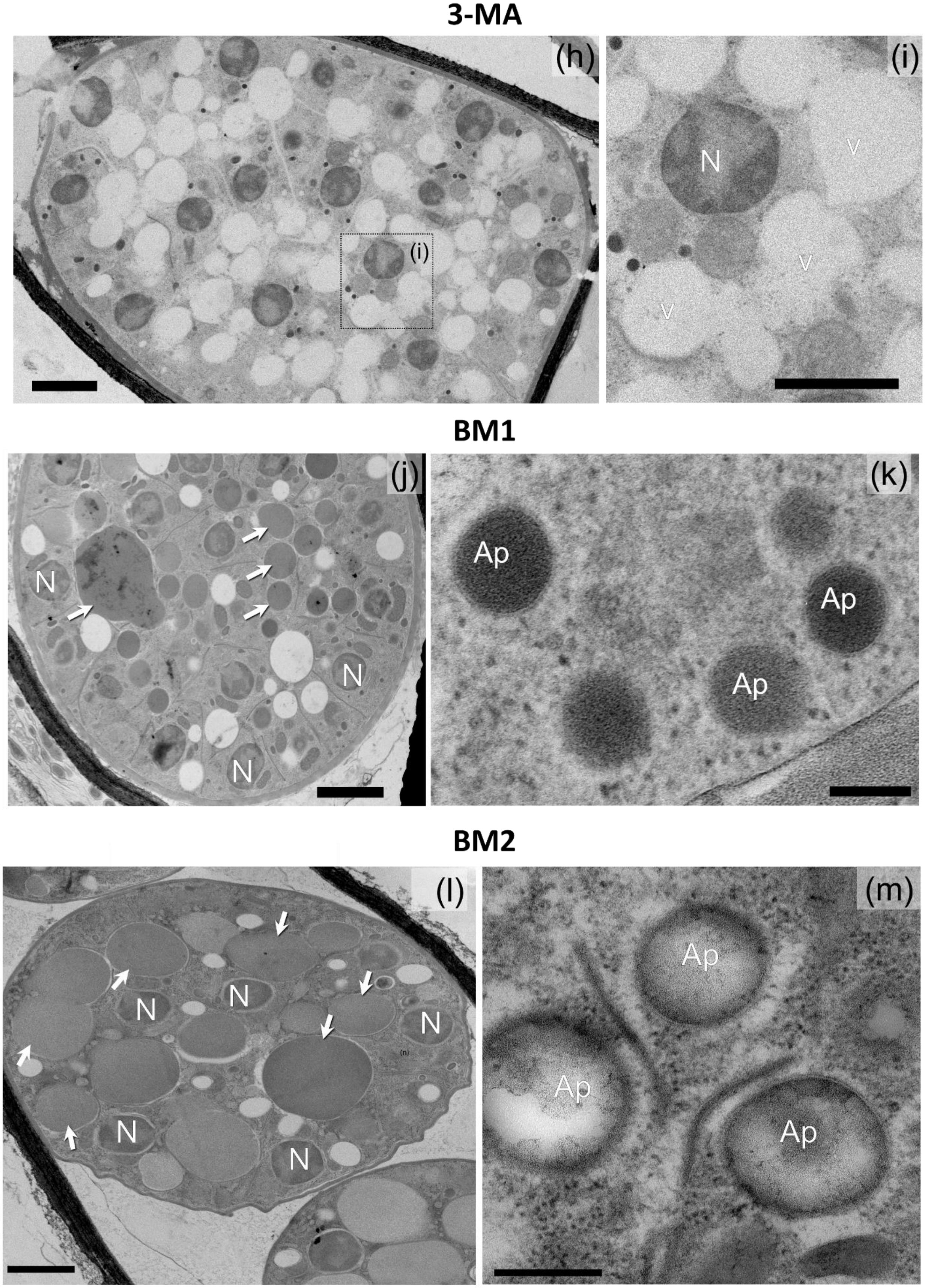
3-MA
(h)
(i)
(i)
N
V
V
V
BM1
(j)
(k)
N
N
N
Ap
Ap
Ap
BM2
(l)
(m)
N
N
N
N
N
N
Ap
Ap
Ap

CQ1

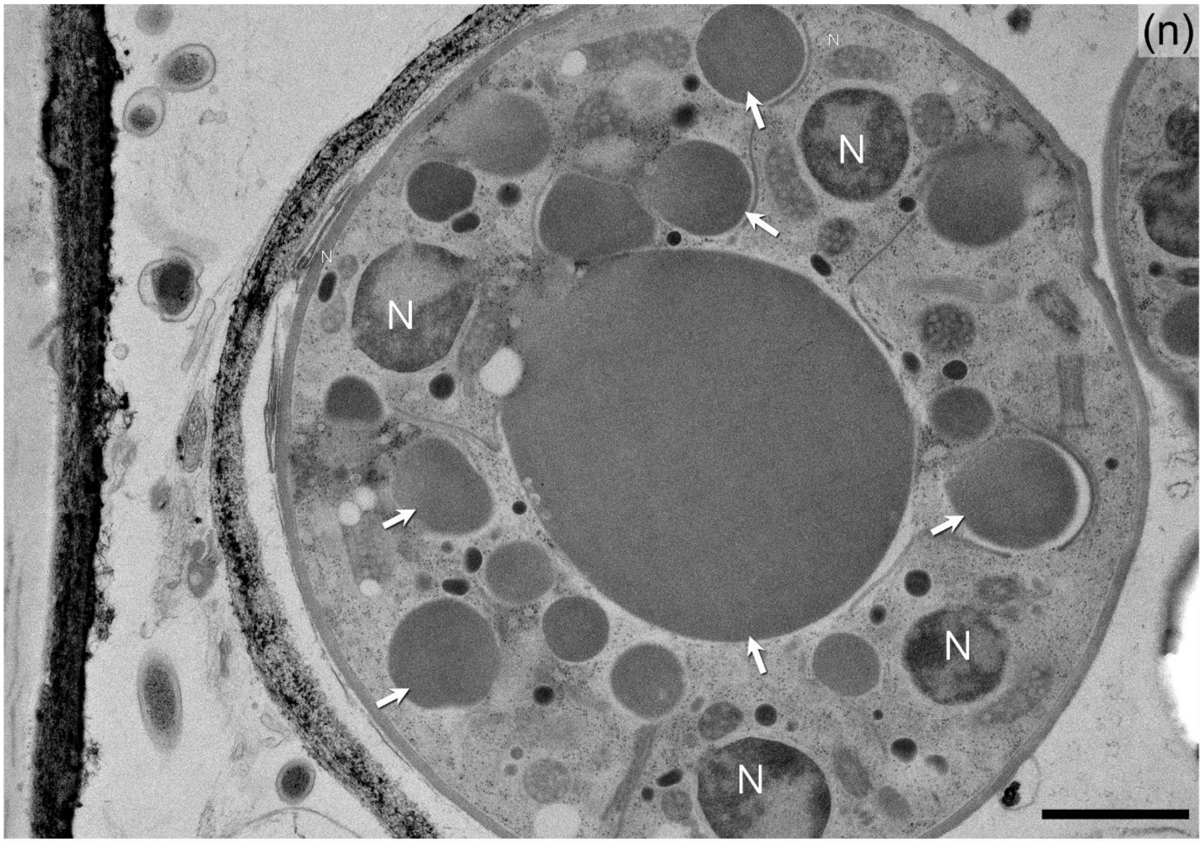

CQ2

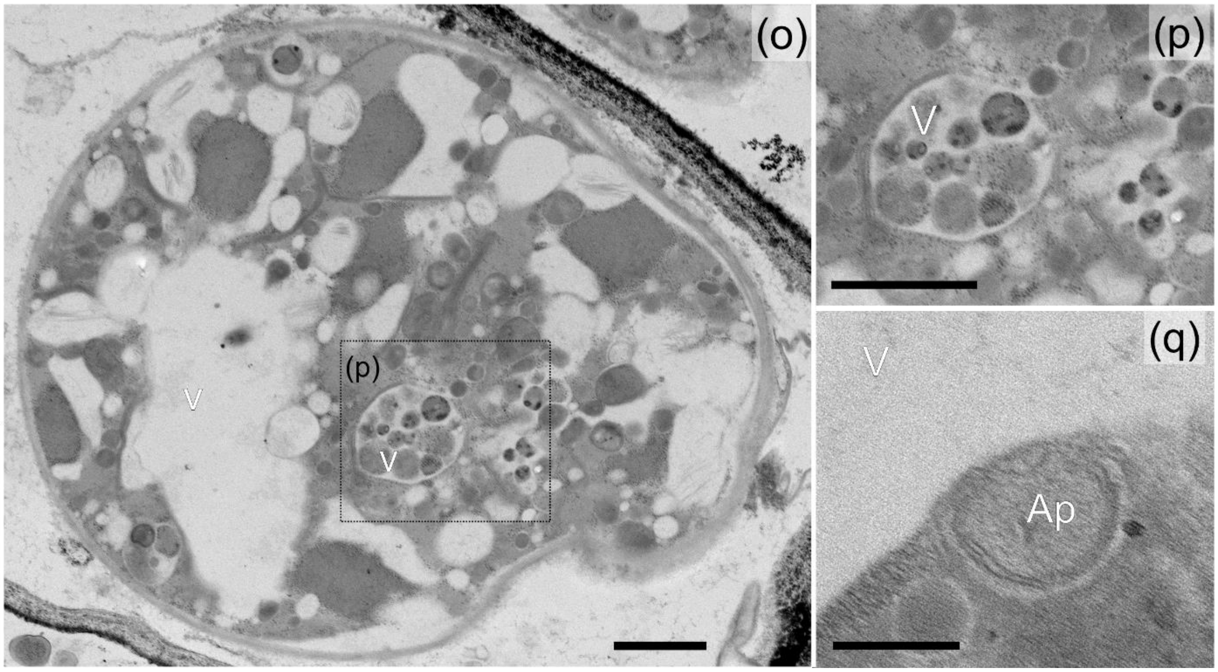

Fig. S7 Representative images illustrating the loss of Lysotracker red signal in *A. ectocarpii* following a 10-day treatment with autophagy inhibitors. A quantification of this response is presented on Fig. 2h. Arrowheads: Lysotracker negative *A. ectocarpii* thallus. Scale bars: 16 µm.

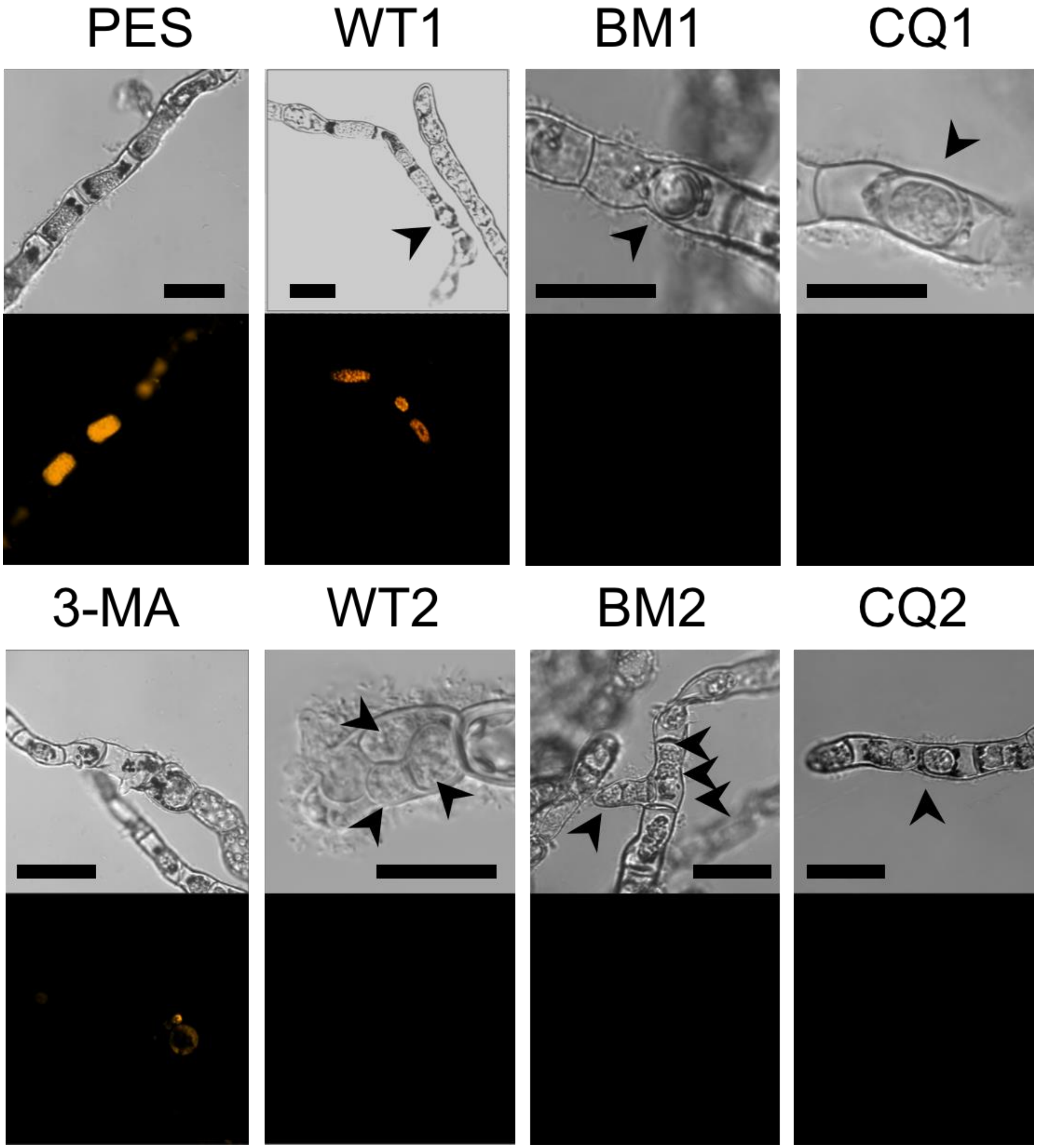

Fig. S8 Progressive loss of MDC signal during a 10-day incubation of *A. ectocarpii* thalli in autophagy inhibitors, using the set-up described in Suppl. Fig. 2c. Representative images of cultures at 10 dai are shown on Suppl. Fig. 5f. Each curve shows the evolution of one biological replicate over time.

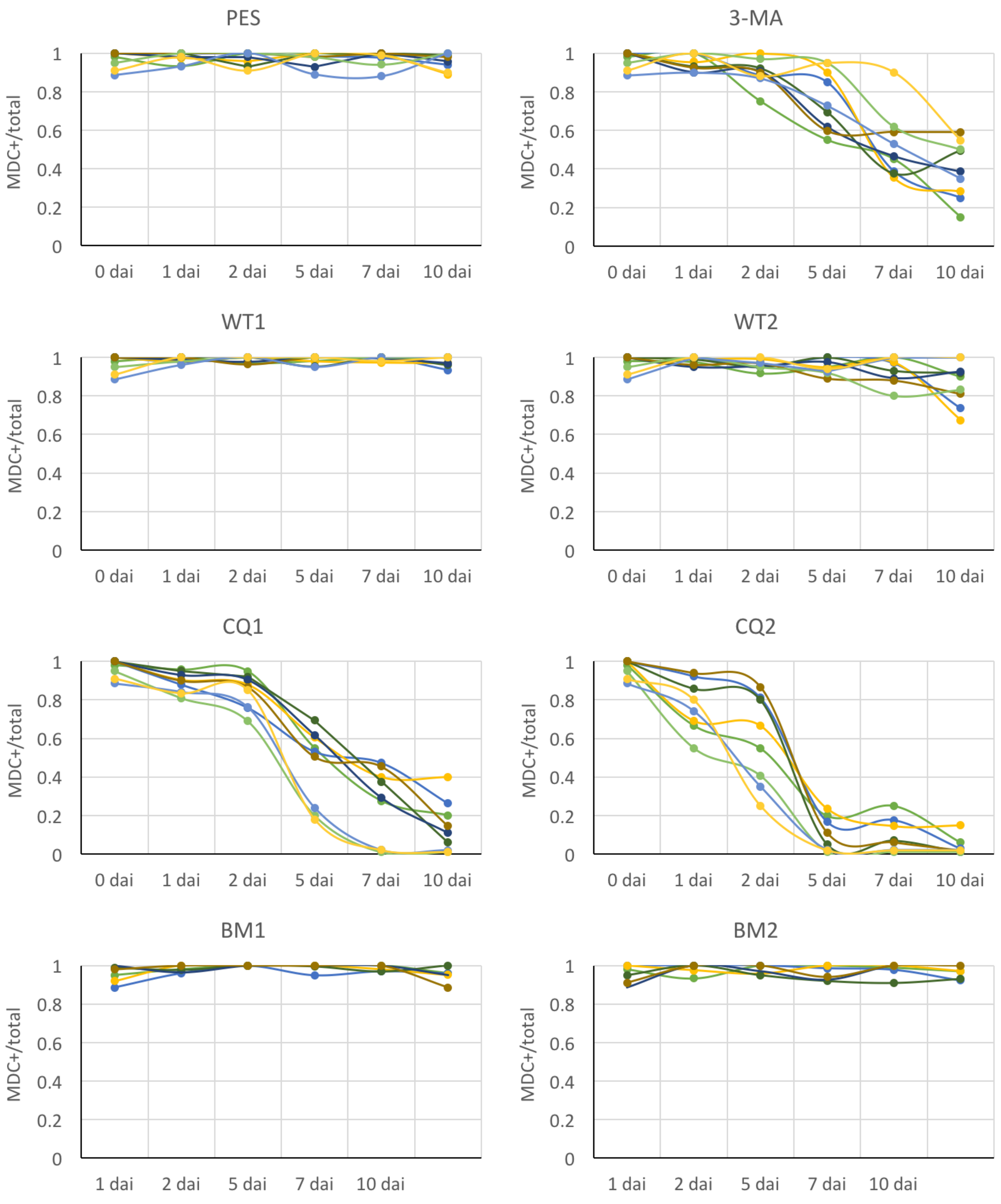

Fig. S9 Host cell wall reinforcement and other cell rearrangements during the *A. ectocarpii* infection course revealed by CHF. (a) Magnification of an infected *M. pyrifera* cell with a collapsed plasmalemma (arrowheads) highlighted as inset on Fig. 3c. N: pathogen nucleus; Cy: pathogen cytosol; CW: pathogen cell wall; HCy: host cytosol; HCW: host cell wall. Scale bar: 2 μm. (b) Magnification of the collapsed plasmalemma region. Arrowhead: early cell wall deposition. Scale bar: 250 nm. (c) The successful deposition of a second cell wall (CW2) keeps the pathogen at bay and allows the cell survival. CW1: old (ante-infection) cell wall. Sp: *A. ectocarpii* spore; P: papilla. Scale bar: 2 μm. (d) Magnification of the periphery of the challenged host cell showing the interface of dividing chloroplasts (arrowheads). Note as well the accumulation of small vacuoles between the plasmalemma and the plastids. Cp: Chloroplasts. Scale bar: 1 μm. (e) Magnification of a central digestive vacuole from Fig. 3g (inset S9e), with unrecognizable cell debris. Scale bar: 500 nm. (f) Magnification of non-host objects boxed as inset S9f on Fig. 3g, which resemble *A. ectocarpii* lipid globules (Lg). Note the presence of a membrane around some of their surface. Scale bar: 500 nm.

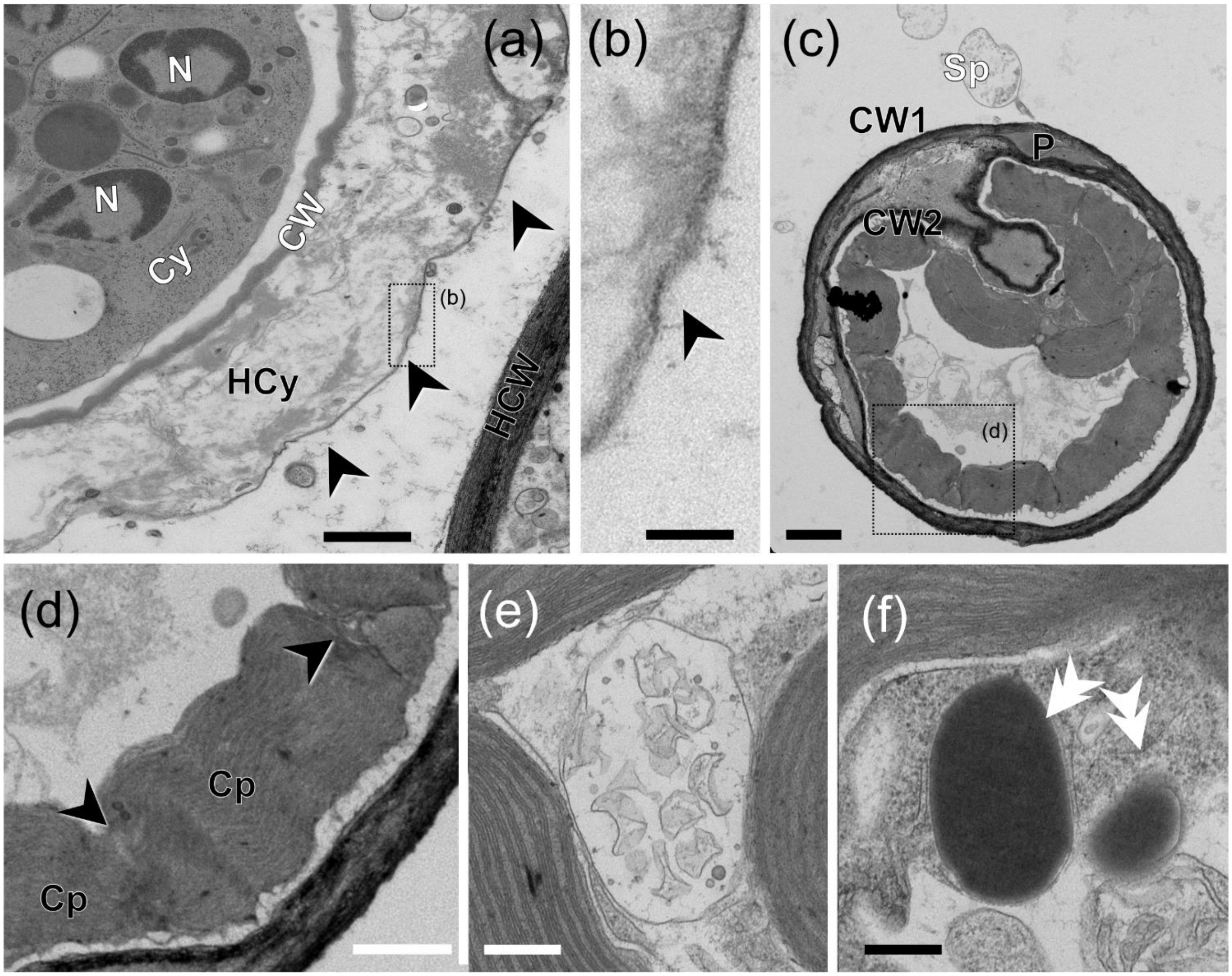

(a)
N
N
Cy
CW
HCy
(b)
HCW
(b)
(c)
Sp
CW1
P
CW2
(d)
(d)
Cp
Cp
(e)
(f)

Fig. S10 Ultrastructure of a mock-challenged *Macrocystis pyrifera* under different TEM techniques. (a) Filament of vegetative *M. pyrifera* cells. V: vacuole. Scale bar: 3 µm. Inset: healthy *M. pyrifera* filament under DIC microscopy. Scale bar: 15 µm. (b) Magnification of the cell periphery, highlighting the normal cell wall thickness (CW) and the plastid morphology (Cp). Scale bar: 500 nm. (c) Appearance of a normal nucleus (N). Scale bar: 500 nm. (d) Morphology of healthy mitochondria (M), neighbouring the plastids. Scale bar: 500 nm. (e) Analogous *M. pyrifera* material to (a) to (d), observed after chemical fixation (CHF). Scale bar: 4 µm. (f-h) Morphology of (f) plastids, (g) mitochondria and (h) nucleus and endoplasmic reticulum (ER). Scale bars: 1 µm (f) and 500 nm (g and h).

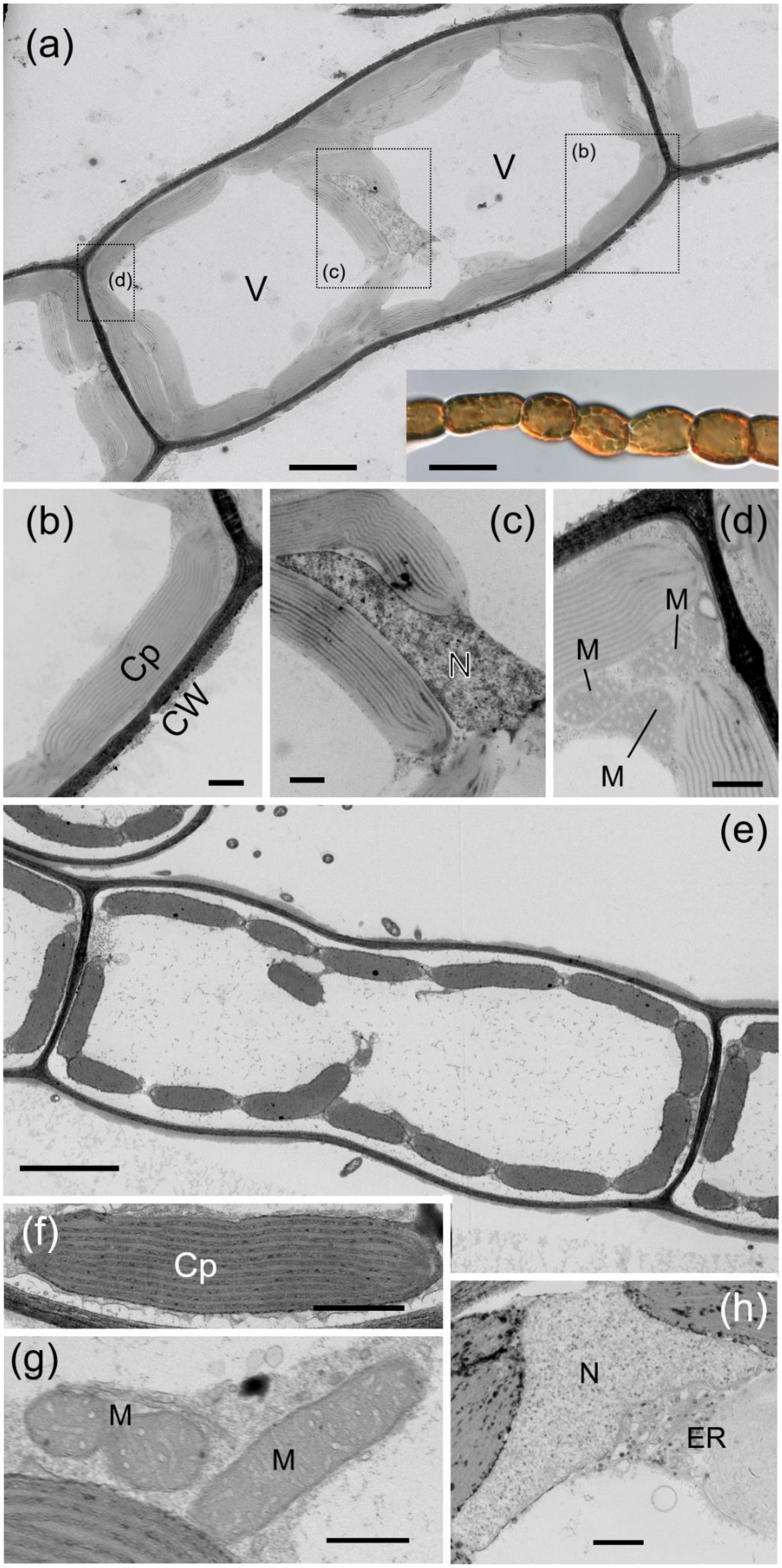

(a)
V
V
(b)
(c)
(d)
(b)
Cp
CW
(c)
N
(d)
M
M
M
(e)
(f)
Cp
(g)
M
M
(h)
N
ER

Fig. S11 Pattern of MDC staining in mock-challenged *M. pyrifera* and the sensitivity of inducible MDC staining in unchallenged *M. pyrifera* cells to the application of autophagy inhibitors. (a) Control, mock-challenged *Macrocystis pyrifera* under DIC and epifluorescence (double staining with MDC and CFW) after 20 days in culture. The MDC staining is weak and limited to very few apical cells (arrowhead) or damaged cells (not shown). Scale bar: 15 µm. (b) Loss of MDC labelling in unchallenged cells from a challenged culture, following treatment with autophagy inhibitors. Whereas healthy unchallenged cells from *Anisolpidium*-challenged cultures ("PES" treatment with no autophagy inhibitors) are commonly MDC-positive (arrowhead in the upper left panel), the MDC signal was virtually absent from unchallenged host cells in the presence of autophagy inhibitors. Arrows point to *A. ectocarpii* thalli, which sometimes may be MDC-positive under these inhibitor treatments (see Suppl. figs. 5 and 8). Scale bars: 16 µm.

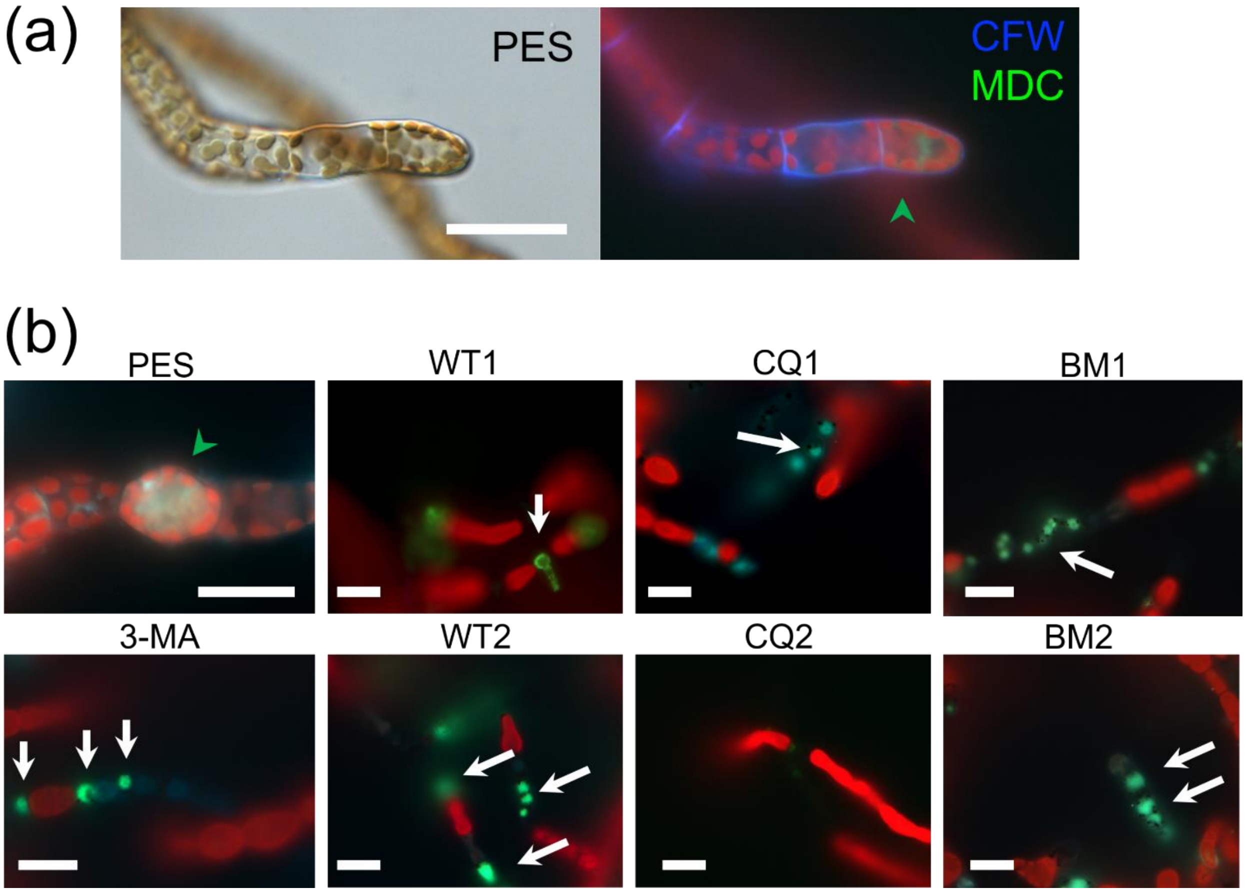

Fig. S12 Sensitivity of Lysotracker staining in unchallenged host cells to the application of autophagy inhibitors. Arrowheads: Lysotracker-positive unchallenged cells in a culture challenged with *A. ectocarpii*. Arrows: Lysotracker positive *Anisolpidium* thalli. After treatment with autophagy inhibitors, equivalent unchallenged host cells from a challenged culture are not Lysotracker positive any more. Scale bars: 16 µm.

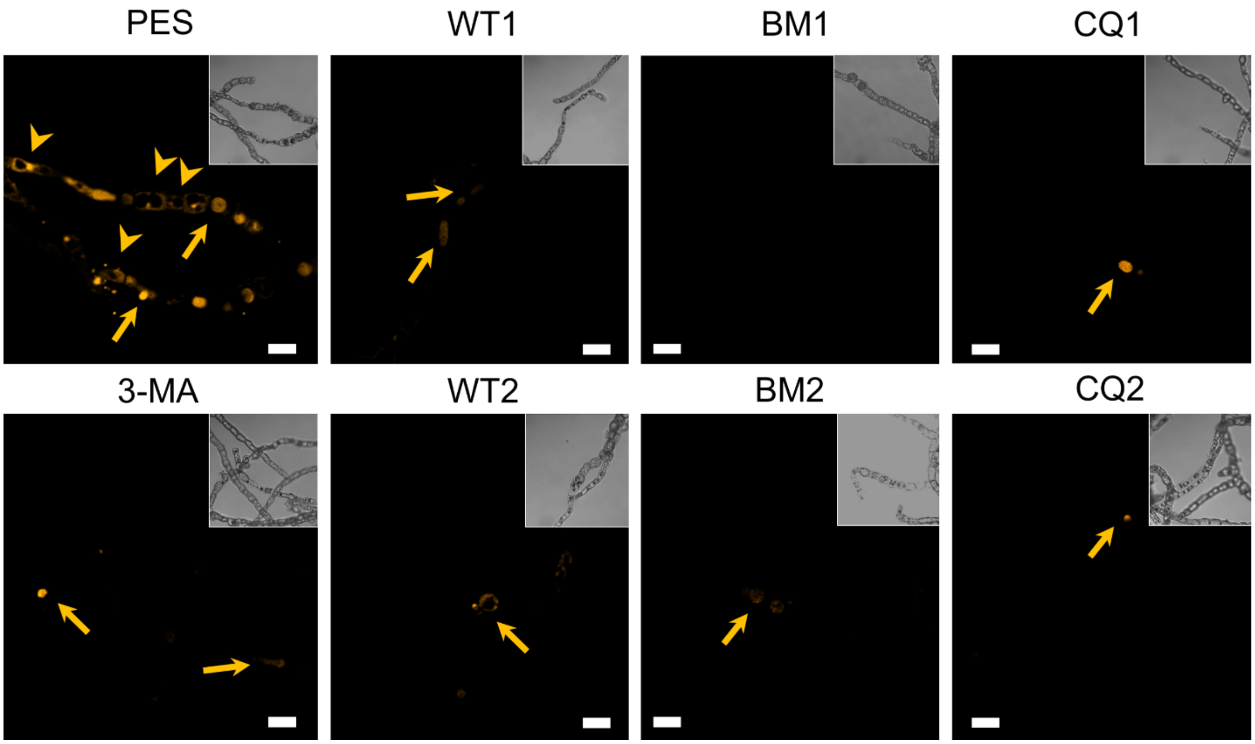

Fig. S13 Ultrastructural changes (CHF) in an unchallenged host cell from a challenged inoculum. (a) Ultrastructural overview of an unchallenged host cell (as judged by the absence of thallus or papilla) that is neighbouring a challenged cell (asterisk, as judged by the presence of a papilla and secondary cell wall). The image shows the formation of peripheral vesicles (arrows), normally absent in mock-challenged controls. HN: Host nucleus; Cp: Chloroplast; GA Golgi apparatus; M: Mitochondrion; Hd: host debris; P: Papilla. Scale bar: 2 μm. (b) Multi-lamellar structures of different size are widely present in such unchallenged cells during *A. ectocarpii* infection. (c) Magnification of a cell boundary from image a (inset (c)), showing vesicles formation in the cell periphery. Scale bar: 1 μm. (d) Magnification of a double-membrane vesicle from image a (inset (d)), filled with unrecognizable cellular elements. GA Golgi apparatus, dv: digestive vesicle. Scale bar: 1 μm.

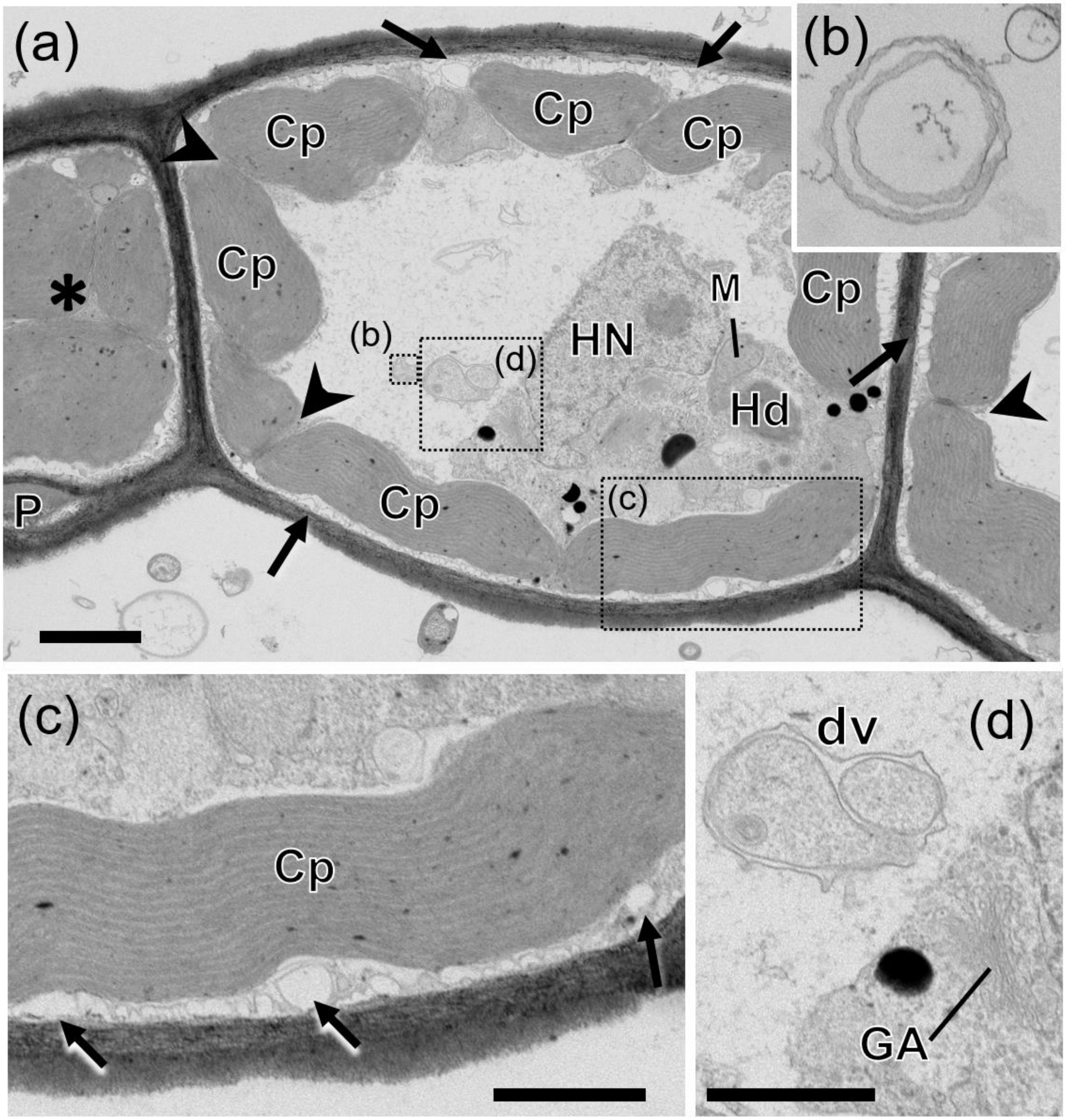

(a)
Cp
Cp
Cp
Cp
*
(b)
(d)
HN
M
Hd
Cp
Cp
(c)
P
(b)
(c)
Cp
dv
(d)
GA

Fig. S14 TEM evidence for plastid division and accumulation under autophagy inhibitor treatments. (a) Comparison of plastid size in control and challenged *Macrocystis* with different autophagy inhibitor treatments, after 10 days incubation using TEM microscopy. PES: Control (Provasoli enriched seawater only); WT1 and WT2: 250 nM and 1 μM Wortmannin; 3-MA: 10 mM 3-Methyladenine; BM1 and BM2: 0.1 and 0.5 μM Bafilomycin. CQ1 and CQ2: 50 and 200 μM Chloroquine. Letter on bars designate the statistical differences using a LMM after Tukey test for multiple comparisons, where $a < b < c$ with $p < 0.001$. (b) Comparison of plastid numbers in control and challenged *Macrocystis* with different autophagy inhibitor treatments after 10 days incubation using TEM microscopy. PES: Control (Provasoli enriched seawater only); WT1 and WT2: 250 nM and 1 μM Wortmannin; 3-MA: 10 mM 3-Methyladenine; BM1 and BM2: 0.1 and 0.5 μM Bafilomycin. CQ1 and CQ2: 50 and 200 μM Chloroquine. Letter on bars designate the statistical differences using a LMM after Tukey test for multiple comparisons, where $a < b < c$ with $p < 0.001$. (c) CHF TEM in the host reveals small plastid are related with an extensive plastid division, a process commonly observed in cell from challenged inocula, but much less common in control *Macrocystis*. Scale bars: 2 μm.

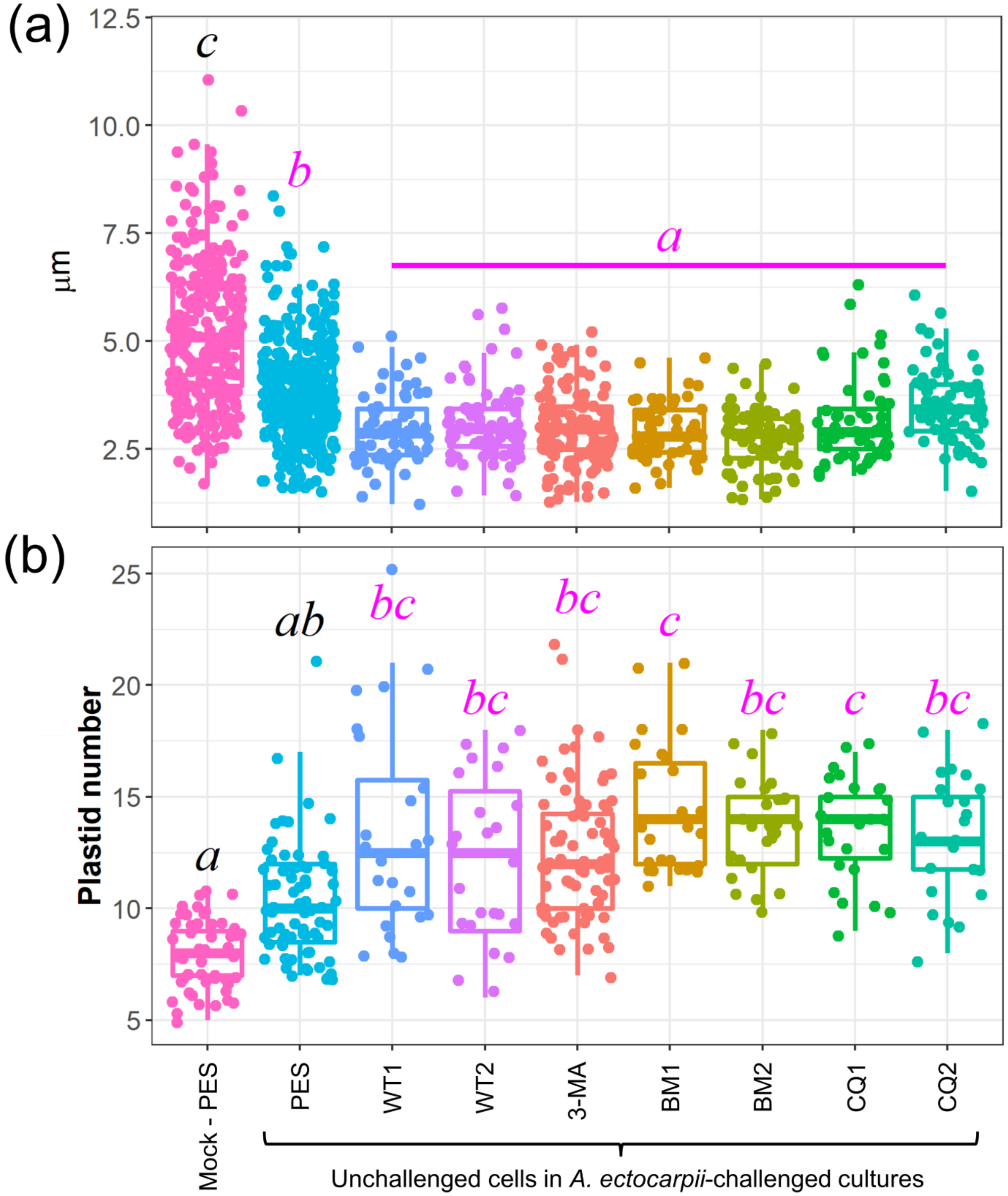

(a)
12.5
10.0
7.5
5.0
2.5
µm
c
b
a
(b)
25
20
15
10
5
Plastid number
a
ab
bc
bc
bc
c
bc
c
bc
Mock - PES
PES
WT1
WT2
3-MA
BM1
BM2
CQ1
CQ2
Unchallenged cells in A. ectocarpii-challenged cultures

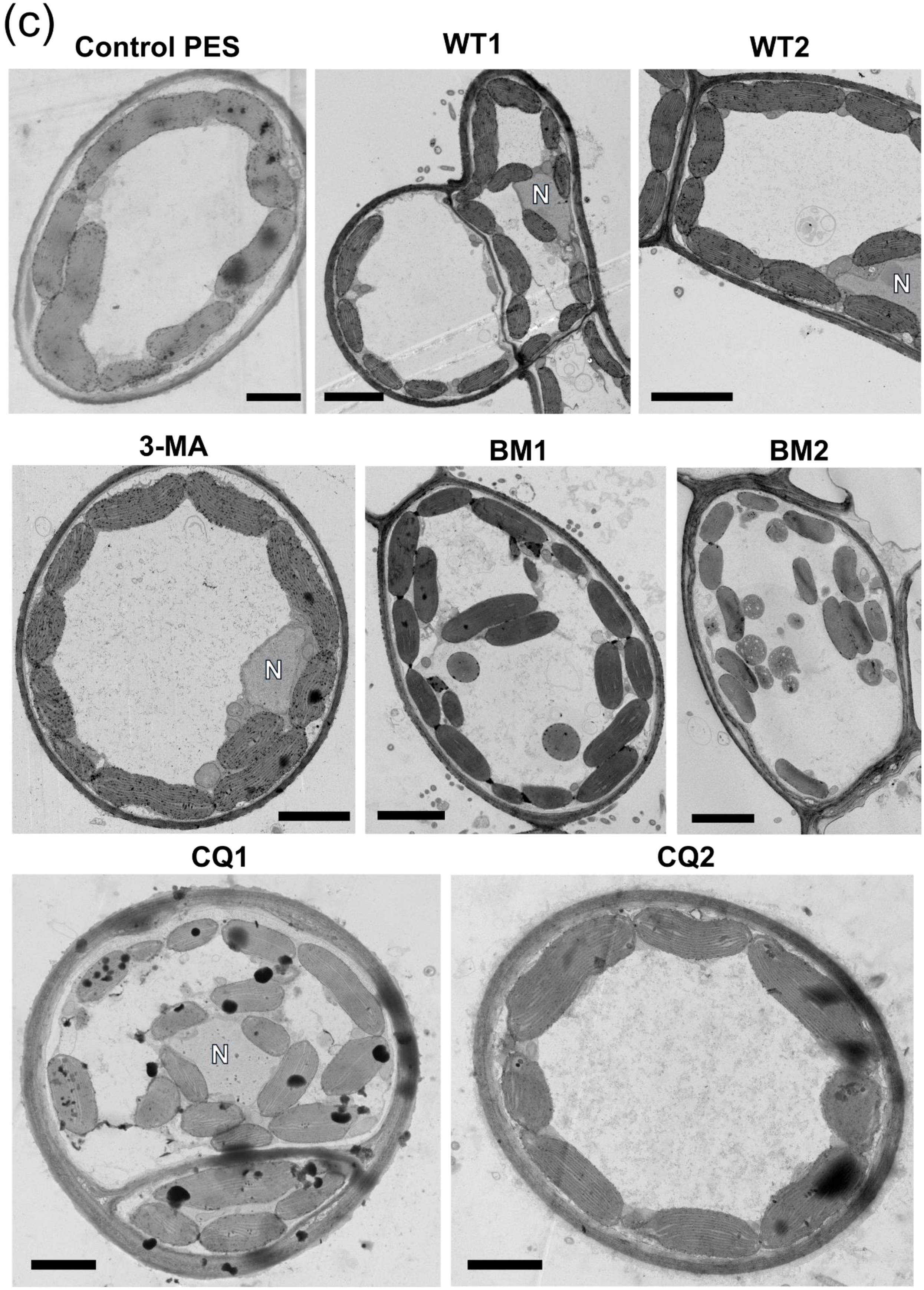
(c)
Control PES
WT1
WT2
N
N
3-MA
BM1
BM2
N
CQ1
CQ2
N

Fig. S15 Vacuolar-plastid interactions resemble potential chlorophagy-like autophagy in challenged *M. pyrifera* inoculum. Coexistence of two potential chlorophagy-like process in *M. pyrifera* cells: (a) whole small plastid (Cp) incorporated and underway to be digested into the vacuole (v) (b) – (c) Vacuolar-plastid junctions resemble piecemeal chlorophagy, mediated by vacuolar junctions (b) or whereby later degradation hints are more evident (c), respectively. v: vacuole. (d) – (e) incorporation (d) and degradation (e) of plastids inside *M. pyrifera* vacuoles of variable size. arrows: digested area. Scale bars: 1 μm

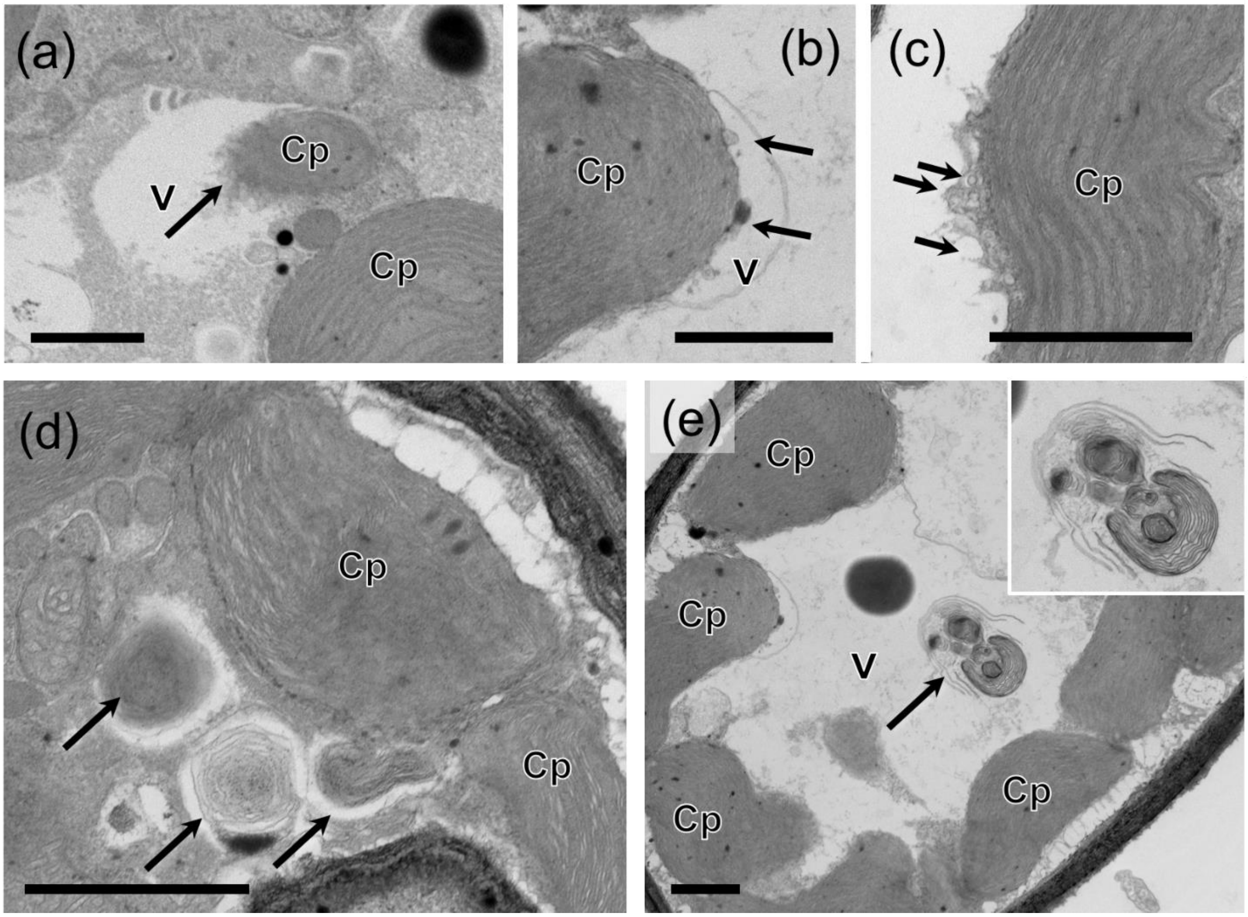

Supplement: Supplementary file 1 — Fig. S1 Development cycle of A. ectocarpii in its host M. pyrifera. Fig. S2 Experimental set‐up for the inoculation of M. pyrifera with A. ectocarpii. Fig. S3 Developmental plasticity of A. ectocarpii syncytia: autophagy regulates sporogenesis in starved thalli. Fig. S4 Lipids accumulate after the exposure to several autophagy inhibitors. Fig. S5 MDC signal is induced in A. ectocarpii during infection, and disrupted after autophagy inhibitor treatments. Fig. S6 Ultrastructural changes undergone by abortive A. ectocarpii thalli following autophagy inhibitor treatments (HPF). Fig. S7 Representative images illustrating the loss of Lysotracker red signal in A. ectocarpii following a 10‐d treatment with autophagy inhibitors. Fig. S8 Progressive loss of MDC signal during a 10‐d incubation of A. ectocarpii thalli in autophagy inhibitors, using the set‐up described in Fig. S2(c). Fig. S9 Host cell wall reinforcement and other cell rearrangements during the A. ectocarpii infection course revealed by CHF. Fig. S10 Ultrastructure of a mock‐challenged M. pyrifera under different TEM techniques. Fig. S11 Pattern of MDC staining in mock‐challenged M. pyrifera and the sensitivity of inducible MDC staining in unchallenged M. pyrifera cells to the application of autophagy inhibitors. Fig. S12 Sensitivity of Lysotracker staining in unchallenged host cells to the application of autophagy inhibitors. Fig. S13 Ultrastructural changes (CHF) in an unchallenged host cell from a challenged inoculum. Fig. S14 TEM evidence for plastid division and accumulation under autophagy inhibitor treatments. Fig. S15 Vacuolar–plastid interactions resemble potential chlorophagy‐like autophagy in challenged M. pyrifera inoculum. [file NPH-226-1445-s001.pdf]
